# FTO O-GlcNAcylation promotes TRIM21-mediated FTO ubiquitination degradation to sustain the negative feedback control of macrophage inflammation

**DOI:** 10.3389/fimmu.2025.1593243

**Published:** 2025-06-26

**Authors:** Lu Zhang, Min Liu, Yan Xie, Bi-Feng Yuan, Zhiyong Peng, Jun Xiong, Xiao-Lian Zhang

**Affiliations:** ^1^ State Key Laboratory of Virology and Biosafety, Department of Immunology Wuhan University Taikang Medical School, School of Basic Medical Sciences, Wuhan University, Wuhan, China; ^2^ Hubei Province Key Laboratory of Allergy and Immunology, Department of Allergy Zhongnan Hospital, Wuhan University, Wuhan, China; ^3^ School of Public Health, Wuhan University, Wuhan, China; ^4^ Department of Critical Care Medicine, Zhongnan Hospital of Wuhan University, Wuhan, China; ^5^ Frontier Science Center for Immunology and Metabolism, Medical Research Institute, Wuhan University, Wuhan, China

**Keywords:** FTO, O-GlcNAcylation, ubiquitination, macrophage, inflammatory response

## Abstract

**Introduction:**

The fat mass and obesity-associated protein (FTO), a key RNA N^6^-methyladenosine (m^6^A) demethylase, has been highlighted for its important role in inflammatory response. Emerging evidences link the O-GlcNAcylation to numerous human diseases, particularly inflammation. However, the specific role and underlying mechanism of FTO O-GlcNAcylation in inflammation remain elusive.

**Methods:**

The FTO O-GlcNAcylation modification was determined by co-immunoprecipitation (Co-IP) assay, metabolic glycan labeling combined with click reaction, and liquid chromatography-tandem mass spectrometry (LC-MS/MS) analysis. Chromatin immunoprecipitation (ChIP)-qPCR and dual-luciferase reporter assay were used to determine FOXO1 binding to the *Gfat*2 promoter and the *Gfat*2 promoter activity during LPS stimulation. The FTO ubiquitination modification and the interaction between FTO and TRIM21 were detected by confocal microscopy, pull-down, and mass spectrometry analysis. The effects of FTO O-GlcNAcylation on the ubiquitination degradation of FTO were assessed by Co-IP and protein stability assays. The *Socs*1 mRNA m^6^A methylation levels were detected by m^6^A-RNA-immunoprecipitation (RIP)-qPCR. The myeloid-specific *Fto* deletion (mye*Fto* ^-/-^) mice, the macrophage depletion and reconstitution experiments, and enzyme-linked immunosorbent assays (ELISA) were used to evaluate inflammatory responses in *Salmonella Typhimurium* (*S. Typhimurium*) or bacterial endotoxin (lipopolysaccharide, LPS) induced sepsis mouse models.

**Results:**

We demonstrate that FTO undergoes O-GlcNAcylation specifically at the serine 95 (Ser95) site. LPS enhances FTO O-GlcNAcylation modification levels by increasing the FOXO1-regulated GFAT2 expression. O-GlcNAcylation of FTO promotes TRIM21-mediated K48-ubiquitination degradation of FTO, which further induces suppressor of cytokine signaling (*Socs*) 1 m^6^A methylation, thus sustains SOCS1 protein expression and suppresses multiple inflammatory cytokines IL-1β/IL-6/TNF-α production in LPS-stimulated macrophages. FTO O-GlcNAcylation mutation (S95A) aggravates *S. Typhimurium* or LPS-induced sepsis, while FTO O-GlcNAcylation suppresses the hyperinflammatory phenotype in mice. Promotion of O-GlcNAcylation by an OGA inhibitor Thiamet-G alleviates LPS-induced inflammatory responses and septic shock in mice.

**Conclusion:**

These findings reveal a mechanism that FTO O-GlcNAcylation promotes its ubiquitination degradation, and thus induces *Socs1* m^6^A methylation and downregulates LPS-mediated inflammatory response, which maintains the negative feedback control of macrophage inflammatory cytokine storm in sepsis. Regulation of FTO O-GlcNAcylation may offer a potential therapeutic strategy for combating endotoxin-induced inflammatory disease and other FTO abnormal expression-associated diseases.

## 1 Introduction

Pathogenic factors such as bacterial endotoxin (lipopolysaccharide, LPS) from Gram-negative bacteria (such as *Salmonella Typhimurium*, *Escherichia coli, Klebsiella pneumoniae*, and *Pseudomonas aeruginosa*) are widely acknowledged for their pivotal roles in initiating inflammatory response and septic shock (1, 2). Sepsis is characterized by multi-organ failure, primarily due to severe systemic inflammation, inflammatory cytokine storm, and dysfunction of host defense mechanisms induced by the deregulation of host defense against infection (3, 4). Sepsis is one of the most fatal diseases worldwide and often induces in-hospital mortality remains higher than 25-30%, and even 40-50% in intensive care units (ICU) when the shock is present (5–7). The activated immune cells (mainly such as macrophages) in the context of sepsis release multiple inflammatory mediators such as interleukin-6 (IL-6), IL-1β, and tumor necrosis factor-α (TNF-α) (8–10). Although there are conventional management interventions for sepsis such as early antibiotics, fluid resuscitation, and hemodynamic support by vasopressors, severe sepsis is still an important cause of death in ICUs worldwide (11, 12). Therefore, there is an urgent need to explore the host molecules involved in inflammation to develop novel effective treatment strategies for sepsis.

The m^6^A demethylase, fat mass and obesity-associated protein (FTO), has been implicated with multiple pathological processes, including diabetes mellitus, liver steatosis, leukemia, melanoma, and other various malignancies (13–18). FTO-mediated m^6^A modification in NOD1 mRNA promotes innate immunity by activating the NOD-like receptor pathway in teleost (19). The GDF6-FTO axis modulates the innate immune and inflammatory response to human respiratory syncytial virus (20). FTO-mediated m^6^A modification alleviates autoimmune uveitis by regulating microglia phenotypes via the GPC4/TLR4/NF-kappaB signaling axis (21). Recently, FTO has been highlighted for its role in promoting inflammation and sepsis (22–26). FTO expression is downregulated by LPS stimulation in macrophages (22–24). LPS treatment induces suppressor of cytokine signaling (*Socs*) 1 m^6^A methylation and sustains SOCS1 induction by decreasing *Fto* mRNA expression, which maintains the negative feedback control of macrophage cytokine storm in sepsis (22). Targeted inhibition of FTO has been shown to protect against LPS-induced septic shock by suppressing NLRP3 inflammasome (24). These previous reports indicated that FTO plays a crucial role in innate immunity and LPS-induced inflammatory diseases. However, the underlying mechanism of FTO downregulation during LPS stimulation needs further clarification.

Emerging evidences link the O-GlcNAcylation to numerous human diseases, particularly inflammatory conditions (27–30). O-GlcNAcylation is an important type of dynamic glycosylation that occurs when a monosaccharide N-acetylglucosamine (GlcNAc) from uridine diphosphate-N-acetylglucosamine (UDP-GlcNAc) is added onto serine (Ser) or threonine (Thr) residues of cytoplasmic and nuclear proteins by O-GlcNAc transferase (OGT) and can be reversibly removed by O-GlcNAcase (OGA) (31, 32). This dynamic O-GlcNAcylation modifies multiple intracellular proteins, including transcription factors, signaling molecules, and metabolic enzymes (32). O-GlcNAc modification is extensively involved in various cellular activities such as the cell cycle, signal transduction, cellular stress response, protein stability, and protein-protein interaction (33–37). Recent studies have unveiled the roles of O-GlcNAcylation in innate immune signaling and inflammation (28, 38). For example, OGT suppresses inflammation and necroptosis by targeting O-GlcNAcylation of RIPK3 (28). However, the role of the potential FTO O-GlcNAcylation during inflammation remains to be further elucidated.

In the present work, we find that FTO undergoes OGT-mediated O-GlcNAcylation and FTO is O-GlcNAcylated at the Ser95 site. LPS does not affect the expression of OGT and OGA, while LPS enhances FTO O-GlcNAcylation by increasing the FOXO1-regulated GFAT2 expression in macrophages. Silence of *Gfat*2 reverses the FOXO1-induced increase in FTO O-GlcNAcylation and LPS enhances FTO O-GlcNAcylation relying on FOXO1-regulated GFAT2 expression in macrophages. FTO O-GlcNAcylation further promotes TRIM21-mediated FTO lysine (K) 48-ubiquitination degradation, which induces *Socs*1 m^6^A methylation, thus sustains SOCS1 protein expression and suppresses multiple inflammatory cytokines IL-1β/IL-6/TNF-α production in LPS-stimulated macrophages. FTO Ser95 site O-GlcNAcylation mutation aggravates *S. Typhimurium* or LPS-induced sepsis, while FTO O-GlcNAcylation suppresses the hyperinflammatory phenotype in mice. Promotion of O-GlcNAcylation by an OGA inhibitor Thiamet-G (TMG) or inhibition of FTO by an FTO inhibitor Meclofenamic acid (MA) alleviates LPS-induced inflammatory responses and septic shock in mice. Taken together, our findings reveal that FTO O-GlcNAcylation promotes its ubiquitination degradation, and thus maintains the negative feedback control of macrophage inflammatory cytokine storm in sepsis. Regulation or enhancement of FTO O-GlcNAcylation might serve as a potential therapeutic strategy for combating endotoxin-induced inflammation or other FTO abnormal expression-associated diseases.

## 2 Materials and methods

### 2.1 Animals

All mouse experiments were carried out in strict adherence to the Chinese National Guideline for Ethical Review of Laboratory Animal Welfare (No. WP20240336). The wild-type (WT) C57BL/6 (20–25 g, 6 to 8 weeks, SPF level, both male and female) mice were approved by the Ethics Committee of the Center for Animal Experiments at Wuhan University. *Ogt* ^fl/fl^ mice were purchased from the Jackson Laboratory (B6.129-*Ogt* ^tm1Gwh^, stock no: 004860). *Fto* ^fl/fl^ mice were provided by Cyagen Biosciences (C57BL/6N-*Fto* ^em1(flox)Cya^, stock no: S-CKO-09550). Mice expressing Cre recombinase (LysM-Cre) were purchased from the Jackson Laboratory (B6.129P2-Lyz2^tm1(cre)Ifo^, stock no: 004781). The Cre-loxP system was used to generate myeloid-specific *Ogt* and *Fto* knockout mice (referred to as *Ogt* ^Δmye^ and mye*Fto* ^-/-^). We generated mye*Fto* ^-/-^ by crossing *Fto* ^fl/fl^ mice with LysM-Cre mice, or *Ogt* ^Δmye^ by crossing *Ogt* ^fl/fl^ mice with LysM-Cre mice. *Fto* ^fl/fl^ and *Ogt* ^fl/fl^ mice serve as control groups. All mice were housed in micro isolator cages, fed with a standard laboratory diet and water at 25°C, and maintained in a 12 h/12 h light/dark cycle.

### 2.2 Cell culture and stimulation

RAW264.7 mouse macrophages and HEK293T human embryonic kidney cells were purchased from the China Center for Type Culture Collection (GDC0143 and GDC0187, respectively) and cultured in Dulbecco’s modified Eagle’s medium (DMEM, Gibco, USA) containing 10% heat-inactivated fetal bovine serum (FBS, Gibco, USA) and 1% penicillin-streptomycin in a humidified incubator with 5% CO_2_ at 37°C. The mouse bone marrow-derived macrophages (BMDMs) were differentiated from bone marrow cells flushed from mouse femurs and tibias by incubation in 10% FBS plus monocyte colony-stimulating factor (M-CSF, 40 ng/mL, Peprotech, USA) for 7 days. Macrophages were stimulated with LPS (1 μg/mL), chloroquine (CQ, 20 μM), 3-methyladenine (3-MA, 5 mM), carbobenzoxy-L-leucyl-L-leucyl-L-leucinal (MG132, 100 nM), cycloheximide (CHX, 500 nM), TMG (20 μM), OSMI-1 (50 μM) or meclofenamic acid (MA, 100 μM) for various periods.

### 2.3 Cell transfection and generation of lentivirus

RAW264.7 and HEK293T cells were subjected to transient transfection using NEOFECT™ DNA transfection reagent according to the manufacturer’s instructions. BMDMs were subjected to si*Gfat2*/si*FoxO1* transfection using Lipofectamine 2000 transfection reagent. The lentiviral vector pCDH-CMV-MCS-EF1a-GFP-puro (SBI, USA) was used to construct pLV*-Fto* (WT)/*Fto* (S95A) expression plasmids. The pLV-*Fto* (WT) or *Fto* (S95A) plasmids were cotransfected into HEK293T cells with the helper vectors pSPAX2 (Addgene, China) and pMD2.G (Addgene, China) using NEOFECT™ transfection reagent. After transfection for 72 h, the cell supernatants were collected and centrifuged at 12,000×g to remove cell debris. The supernatants were transferred to an ultracentrifuge tube, and 2 mL of a 20% sucrose solution was gently added to the bottom of the tube and centrifuged at 25,000×g for 2 h. The supernatants were discarded, and 100 μL of ice-cold PBS was added to resuspend the pellet to harvest the lentivirus. A lentivirus containing only green fluorescence protein (GFP) was used as a negative control (LV-Ctrl). For lentivirus infection experiments, BMDMs were infected with lentivirus LV-*Fto* (WT) or *Fto* (S95A) or control LV-Ctrl with the multiplicity of infection (MOI): 6×10^8^ for 48 h.

### 2.4 Reverse transcription-quantitative real-time polymerase chain reaction

Total RNAs from BMDMs were extracted using TRIzol reagent (Invitrogen, USA). First-strand cDNAs were synthesized using a ReverTra Ace qPCR RT kit (Toyobo, Japan). Conventional PCR was carried out in a BioRad DNA Engine (BioRad, USA). The RT-qPCR reactions were run on an ABI StepOnePlus Real-Time PCR System (Applied Biosystems, USA) using the standard cycling conditions. Target gene expression levels were normalized based on glyceraldehyde-3-phosphate dehydrogenase (GAPDH), using an SYBR Green Realtime PCR Master Mix kit (Toyobo, Japan). Relative RNA levels were calculated by the comparative cycle threshold (CT) method (2^−ΔΔCT^ method), where CT indicates the amplification cycle number at which the fluorescence generated within a reaction rises above a defined threshold fluorescence, and ΔΔCT = experimental sample (Ct _target gene_ − Ct _GAPDH_) − control sample (Ct _target gene_ − Ct _GAPDH_). PCR primers are listed in the **Supplementary Table 1**.

### 2.5 Western blot analysis

RAW264.7, HEK293T or BMDMs were lysed in cell lysis buffer (radioimmunoprecipitation assay, RIPA) containing 10 mM Hepes (pH 7.5), 50 mM NaCl, 1% Triton X-100, and 2 mM EDTA. Protein lysates were separated by SDS**–**PAGE and then electroblotted to polyvinylidene difluoride (PVDF) membranes (Millipore, USA). The membranes were blotted with primary antibodies purchased commercially, followed by incubation with horseradish peroxidase (HRP)-conjugated secondary antibodies. Protein bands were visualized using chemiluminescence by an ECL WB kit (Millipore, Belmopán, Belize). Primary antibodies used for WB analyses are listed in **Supplementary Table 2**.

### 2.6 Coimmunoprecipitation

RAW264.7 or HEK293T cells transfected with plasmids or siRNAs or LPS/GlcN-treated BMDMs were lysed in RIPA lysis buffer with the protease inhibitor phenylmethylsulfonyl fluoride (PMSF). The cell lysates were incubated with the indicated antibodies plus protein-A/G magnetic beads followed by extensive washing with RIPA lysis buffer. The immunoprecipitates were analyzed by SDS**-**PAGE and blotted with the indicated antibodies listed in **Supplementary Table 2**.

### 2.7 Detection of FTO O-GlcNAcylation using metabolic glycan labeling and click chemistry reaction

The RAW264.7 cells were seeded at a density of 1 × 10^6^ cells per mL in culture medium with or without GalNAz (100 μM) and grown at 37°C and 5% CO_2_ for 24 h, with sugar metabolic incorporation into the corresponding cellular GlcNAcylated proteins. The cells were washed three times and lysed using RIPA lysis buffer supplemented with protease inhibitor PMSF. The cell lysates were immunoprecipitated with anti-FTO and protein-A/G magnetic beads, followed by incubation with 50 μM biotin-DBCO (Click Chemistry Tool, USA) at 37°C for 30 min, and then subjected to SDS-PAGE and detection by chemiluminescence using HRP-streptavidin.

### 2.8 Confocal microscopy analysis

To analyze the colocalization of FTO with OGT or TRIM21, RAW264.7 cells (3×10^5^) were seeded in confocal dishes (NEST Biological Technology Co., Ltd., Shanghai, China). After LPS, TMG, or OSMI-1 treatment, the cells were fixed with 4% paraformaldehyde, permeabilized with 0.3% Triton X-100, and blocked with 10% BSA. The cells were then incubated with the indicated antibodies and fluorescence-labeled secondary antibodies. The nuclear staining was mounted with DAPI, followed by washing three times with 1× cold PBS (pH 7.4). The localization of proteins was visualized with a Leica SP2 confocal system (Leica Microsystems, Wetzlar, Germany).

### 2.9 Mapping of FTO O-GlcNAcylation sites

LC-MS/MS was performed to identify the FTO O-GlcNAcylation modification sites. In brief, RAW264.7 cells were transfected with Flag-tagged full-length or N-terminal fragment FTO and treated with TMG. The cell lysates were incubated with the anti-Flag antibody plus protein-A/G magnetic beads followed by extensive washing with RIPA lysis buffer and subjected to SDS**-**PAGE. The corresponding gel bands containing full-length and N-terminal FTO were stained with Coomassie blue, and then the corresponding bands were excised, for LC-MS/MS analysis (Shanghai OmicSolution Co., Ltd, Shanghai, China). The peptides were analyzed by Orbitrap fusion lumos coupled to an EASY-nanoLC 1200 system (Thermo Fisher Scientific, MA, USA). Peak lists were analyzed using Sequest against a murine UniProt database. Tandem mass spectra were processed by PEAKS Studio version 10.6 (Bioinformatics Solutions Inc., Waterloo, Canada). PEAKS DB was set up to search the database of targets assuming trypsin as the digestion enzyme. PEAKS DB was searched with a fragment ion mass tolerance of 0.02 Da and a parent ion tolerance of 7 ppm. Carbamidomethylation (C) was specified as the fixed modification. Oxidation (M), Deamidation (NQ), Acetylation (Protein N-term), and N-acetylhexosamine (HexNAc, +203.0794 Da) residues were specified as the variable modifications. The peptides with ^-10^lgP ≥ 20 and containing at least 1 unique peptide were filtered.

### 2.10 Determination of UDP-GlcNAc content in BMDMs by high-performance liquid chromatography-tandem mass spectrometry

To detect the effect of LPS stimulation or *Gfat2* silencing on the levels of UDP-GlcNAc, HPLC-MS was performed. Approximately 5 × 10^6^ BMDMs treated with LPS or transfected with si*Gfat2* were resuspended in 500 μL methanol (80%, precooled at −80°C), incubated for 20 min at −80°C, and centrifuged at 12000 × g for approximately 40 min to remove the sediment. Supernatants were lyophilized, resuspended in ice-cold water, and subjected to detection of UDP-GlcNAc levels by HPLC**–**MS system equipped with a Hypercarb PGC column (Thermo Fisher Scientific, USA) coupled by ESI to a QTRAP 6500 (AB SCIEX, USA). In brief, samples and a series of standard UDP-GlcNAc solutions were filtered at 0.22 μm. The column was maintained at 60°C and samples (10 mL volume) were injected at a constant flow rate of 150 mL/min. The binary mobile phase was composed of 0.1% formic acid adjusted to pH 9 with ammonia (A) and acetonitrile (B). The LC run started with a gradient from 2% to 15% acetonitrile for 30 min, ramping linearly to 50% for 42 min, and followed by a gradient to 100% acetonitrile within 1 min, which was maintained for 2 min. The levels of UDP-GlcNAc were quantified by extrapolating the calibration curve constructed from a known amount of commercial sugar nucleotides based on the ratio between metabolite and internal standard MS signals.

### 2.11 Chromatin immunoprecipitation-qPCR

To determine whether the transcription factor FOXO1 binds to the *Gfat*2 gene promoter, ChIP-qPCR was performed. Approximately 1 × 10^7^ BMDMs were treated with or without LPS for 12 h. The DMEM media was removed, and the cells were rinsed three times with cold PBS. Then, the cells were treated with formaldehyde to a final concentration of 1% at room temperature for 10 min. Glycine was added to the cells to a final concentration of 0.125 M to stop the crosslinking reaction. Cells were harvested into cold PBS by scraping and transferred to a 1.5 mL microcentrifuge tube. After centrifugation at 1000 × g for 5 min at 4 °C, the formaldehyde-crosslinked cells were collected and resuspended in 1 mL of nuclear lysis buffer (50 mM Tris-HCl pH 8.0, 10 mM EDTA pH 8.0, 1% SDS, and 1 mM PMSF). Chromatin was sheared to an average size of 100-500 bp by sonication and then centrifuged (10 min, 10,000 × *g*, 4 °C). 60 μL of supernatants were diluted 10-fold with 540 μL of ChIP dilution buffer (1% Triton X-100, 1.2 mM EDTA, 167 mM NaCl, and 16.7 mM Tris-HCl, pH 8.0) and then incubated with anti-FOXO1 or anti-rabbit IgG overnight at 4 °C with rotation. Then, 50 μL of Protein-A/G magnetic beads were added to each sample, and the incubation continued at 4°C for 5 h on a rotating platform. The beads were pelleted and then washed sequentially with low-salt buffer (150 mM NaCl, 20 mM Tris-HCl pH 8.0, 0.1% SDS, 0.5% Triton X-100, and 2 mM EDTA), high-salt buffer (0.1% SDS, 1% Triton X-100, 2 mM EDTA, 20 mM Tris-HCl, pH 8.1, and 500 mM NaCl), and LiCl buffer (0.25 M LiCl, 1% sodium deoxycholate, 10 mM Tris-HCl pH 8.0, 1% NP-40, and 1 mM EDTA), followed by two washes with TE buffer (1 mM EDTA and 10 mM Tris-HCl, pH 8.0). Chromatin was eluted from the beads by two incubations with 100 μL of elution buffer (100 mM NaHCO_3_ and 1% SDS), the Na^+^ concentration was adjusted to 300 mM with 5 M NaCl, and the crosslinks were reversed by an overnight incubation in a 65°C water bath. The samples were then incubated with 0.1 mg/mL RNase at 37°C for 1 h followed by incubation with 1 mg/mL proteinase K at 55°C for 2 h. Captured DNA was purified with a DNA Purification Kit (DP214, TIANGEN, Beijing) and then used for qPCR. The primers targeting the promoter site of the *Gfat2* were synthesized from TSINGKE (Wuhan, China) and listed in **Supplementary Table 1**.

### 2.12 Dual-luciferase reporter assay

For *Gfat*2 promoter activity detection, RAW264.7 cells were cotransfected with a firefly luciferase reporter plasmid comprising the -500 bp region upstream of the transcription start site of the mouse *Gfat2* gene (pGL3-*Gfat2*-promoter-luc), *Gfat2*-luciferase reporter mutant constructs, a Renilla luciferase reporter expression plasmid, and pcDNA3.1-Myc-His-FOXO1/empty vector pcDNA3.1-Myc-His, respectively, and then stimulated with or without LPS for 12 h. Luciferase activity was measured using the Dual-Luciferase Reporter Assay System according to the manufacturer’s instructions (Promega, United States). Data are normalized for transfection efficiency by dividing firefly luciferase activity by Renilla luciferase activity.

### 2.13 Liquid chromatography-tandem mass spectrometry

To detect the effect of FTO/OGT overexpression on the levels of m^6^A levels in RAW264.7 cells, LC-MS/MS was performed. RNAs from RAW264.7 cells-transfected with Flag-*Fto*/Myc-*Ogt* were extracted using a TRIzol reagent. 50 ng of mRNAs were enriched from above-extracted RNAs using VAHTS mRNA capture beads and digested by nuclease P1 (1 U) in 25 µL of buffer containing 10 mM NaCl and 2 mM of ZnCl_2_ at 37°C for 2 h, followed by the addition of NH_4_HCO_3_ (1 M, 2.5 µL, freshly made) and shrimp alkaline phosphatase (SAP, 1 U) and additional incubation at 37°C for 2 h. Samples were centrifuged at 12 000 × g at room temperature for 20 min; 3 µL of the solution was loaded into the Shimadzu LC**-**MS/MS system (LCMS‐8050, Shimadzu, Japan). Nucleosides were quantified by using retention time and nucleoside-to-base ion mass transitions of 282.1 to 150.1 (m^6^A) and 268 to 136 (A).

### 2.14 FTO pull-down assay and mass spectrometry analysis

To investigate FTO-interacting partner proteins, approximately 5 × 10^6^ BMDMs with or without LPS treatment for 12 h were lysed in RIPA lysis buffer with PMSF. The cell lysates were incubated with the anti-FTO antibody plus protein-A/G magnetic beads followed by extensive washing with RIPA lysis buffer. The eluted proteins were analyzed by SDS-PAGE. The specific bands were excised and subjected to LC-MS (TripleTOF 5600+, SCIEX, USA). The data files generated were searched with ProteinPilot version 4.5 (AB SCIEX Foster City, CA, USA) using the Paragon™ algorithm against the UniProt mouse reference proteome database. Iodoacetamide of Cys residues was selected as a fixed modification. An Unused Score cut‐off was set to 1.3 (95% confidence for identification). The protein peak areas were normalized to the total peak area of the respective sample. Proteins with absolute fold change ≥ 1.5 are highlighted as differentially expressed proteins.

### 2.15 m^6^A-RNA-immunoprecipitation-qPCR

For the RIP assay, LV*-*Ctrl/*Fto* (WT)/*Fto* (S95A)-infected mye*Fto* ^-/-^ mouse BMDMs were treated with LPS (1ug/ml) for 12 h, and then lysed in polysome lysis buffer [100 mM KCl, 10 mM HEPES (pH 7.0), 0.5% NP40, 5 mM MgCl_2,_ 1 mM DTT, 80 U/ml RNase inhibitors, and the protease inhibitor phenylmethylsulfonyl fluoride (PMSF)] for 10 min on ice. Cell lysates were sonicated and centrifuged to obtain fragment chromatin and RNA. The fragmented chromatin and RNA were incubated with rabbit anti-m^6^A antibody conjugated protein-A/G magnetic beads, or IgG control overnight at 4°C. Before incubation, 1/10 of the supernatant was set aside to be used as input. After incubation, the samples were washed 5 times with NT buffer [50 mM, Tris-HCl (pH 7.4), 1 mM MgCl_2_, 150 mM NaCl, and 1% Triton X-100]. Immunoprecipitated RNAs and input RNAs were extracted using TRIzol reagent, digested with RNase-Free DNase Set (Qiagen, Germany) to remove any residual DNA, and analyzed by qPCR.

### 2.16 Enzyme-linked immunosorbent assay for cytokine measurement

The wild-type/*Fto* ^fl/fl^/mye*Fto* ^-/-^ mouse BMDMs were infected with LV*-*Ctrl/*Fto* (WT)/*Fto* (S95A) for 48 h and treated with LPS for 12 h. Supernatants were harvested, and the levels of cytokines were evaluated using ELISA by measurement of OD_450_. The TNF-α/IL-1β/IL-6 of the BMDM supernatants were determined using mouse ELISA kits (Dakewe Biotechnology, Beijing, China).

### 2.17 Mouse models for LPS or *S. Typhimurium*-induced sepsis

For LPS-induced sepsis in macrophage-specific *Fto* knockout mice, *Fto^fl/fl^/Fto* ^Δmye^ mice were intraperitoneally (*i.p.*) administered with 25 mg/kg LPS dissolved in PBS (pH 7.0), and then monitored for up to seven days. Control mice were injected with 0.05 mL PBS for each mouse. To evaluate serum cytokines production in mice, one-day post LPS (25 mg/kg) injection, mice were euthanized, and the freshly isolated serum TNF-α/IL-1β/IL-6 concentrations were determined by ELISA immediately.

For mouse macrophage depletion and reconstitution, C57BL/6 mice were intravenously (*i.v.*) injected with clodronate liposomes (CLs) or liposome controls (0.2 mL/mouse, 5 mg/mL of CLs) (39). Elimination of F4/80^+^ macrophages was confirmed by flow cytometry (FCM) at 48 h post-injection, eliminating approximately 90% of F4/80^+^ macrophages. Two days after the clodronate-liposome treatment, macrophage-depleted mice were reconstituted with 2×10^6^ BMDMs per mouse (M-CSF stimulation for 5 days, dissolved in 0.2 mL PBS), through intravenous injection. The reconstituted mye*Fto* ^-/-^ BMDMs had been rescued with LV-Ctrl/*Fto* (WT)/*Fto* (S95A), and wild-type BMDMs had been treated with DMSO/MA (100 μM)/TMG (20 μM) for 24 h.

Two days after adoptive transfer, these mice were intraperitoneally injected with LPS (20/25 mg/kg) or PBS (pH 7.0) or intragastrically (*i.g.*) infected with either a lethal (2×10^7^ cfu) or a sublet hal (2×10^6^ cfu) dose of *S. Typhimurium*, and then monitored for up to seven days. One day post LPS injection, mice were euthanized, and the freshly isolated serum TNF-α/IL-1β/IL-6 concentrations were determined by ELISA. The lethal dose groups (n=8 per group) were used for observing survival rates while the sublethal dose groups (n=3 per group) were used for colon detection. The colon tissues from the sublethal dose groups were harvested after *S. Typhimurium* infection for 72 h.

### 2.18 Hematoxylin and eosin staining

Mouse colon tissue samples were collected and immobilized in 4% paraformaldehyde, embedded in paraffin. Paraffin slides were dewaxed and hydrated as previously reported (40). The dewaxed sections were stained with hematoxylin solution for 10 min, washed with tap water, and soaked in differentiation solution for 30 sec. The sections were then transferred to warm water at 50°C for 5 minutes, stained with eosin solution for 1 minute, and rinsed with tap water. Sections were dehydrated with a graded series of alcohol solutions and cleared in xylene.

### 2.19 Statistical analysis

All statistical analyses were performed using GraphPad Prism 8. For comparison between two mean values, a two-sided unpaired Student’s t-test was used to calculate statistical significance. For comparisons among multiple groups, one-way ANOVA followed by Dunnett’s multiple comparisons tests, or two-way repeated measures ANOVA followed by Tukey’s multiple comparisons tests were used. *p* value < 0.05 is considered to be statistically significant (*p < 0.05, **p < 0.01, ***p < 0.001, ****p < 0.0001).

## 3 Results

### 3.1 FTO undergoes OGT-mediated O-GlcNAcylation

**Figure 1:**
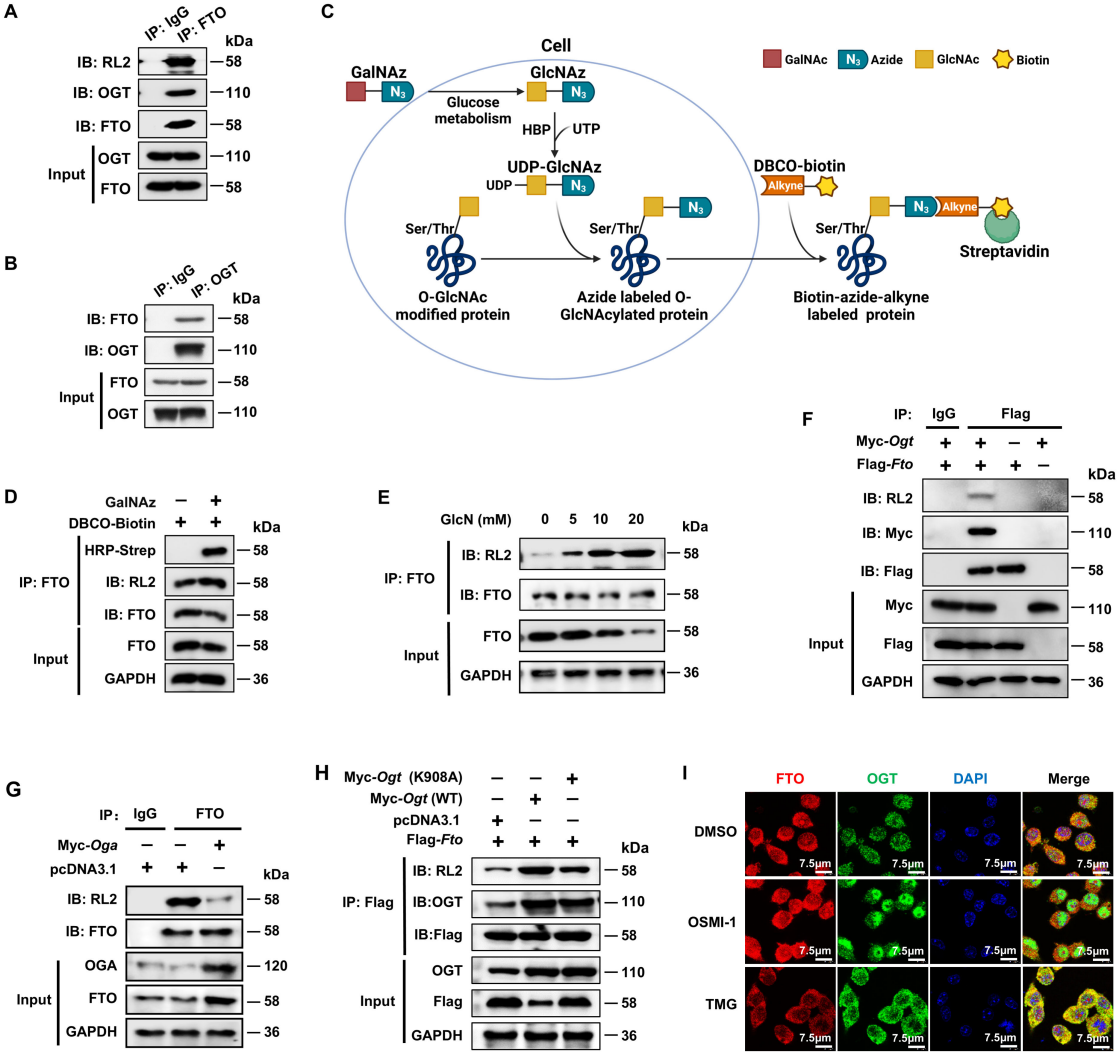
FTO undergoes OGT-mediated O-GlcNAcylation. **(A, B)** Co-IP and immunoblot analysis of O-GlcNAcylated-FTO interacting with OGT in BMDMs. BMDM cell lysates were subjected to Co-IP with an anti-FTO **(A)** or anti-OGT **(B)** and immunoblot with O-GlcNAc-specific anti-RL2, anti-OGT, and anti-FTO, respectively. **(C)** Schematic for GalNAz tagged azide-alkyne bioorthogonal click chemistry. **(D)** The RAW264.7 cells were incubated with GalNAz for 24 h, then the cells were lysed. The total cellular proteins were immunoprecipitated with an anti-FTO antibody and protein-A/G magnetic beads, followed by incubation with DBCO-biotin, and then subjected to SDS-PAGE and detection by chemiluminescence using HRP-streptavidin and anti-RL2, respectively. **(E)** Co-IP and immunoblot analysis of the effect of GlcN on FTO O-GlcNAcylation in BMDMs. BMDMs were incubated with GlcN with different concentrations for 24 h and then subjected to Co-IP with an anti-FTO Ab and immunoblot with Abs against FTO and RL2. **(F)** Co-IP and immunoblot analysis of the interaction between exogenous FTO and OGT. HEK293T cells were transfected with Flag*-Fto* and Myc-*Ogt* for 48 h and subjected to Co-IP with an anti-Flag and immunoblot with Abs against RL2, Myc and Flag, respectively. **(G)** Co-IP and immunoblot analysis of the effect of OGA on FTO O-GlcNAcylation. HEK293T cells were transfected with Myc-*Oga* for 48 h and subjected to Co-IP with an anti-FTO and immunoblot with Abs against RL2, OGA, and FTO, respectively. **(H)** Co-IP and immunoblot analysis of the effect of OGT on FTO O-GlcNAcylation. HEK293T cells were transfected with Flag*-Fto* and Myc-*Ogt* (WT)/(K908A) for 48 h and subjected to Co-IP with an anti-Flag and immunoblot with Abs against RL2, OGT and Flag, respectively. **(I)** Immunofluorescence image of FTO co-localization with OGT upon treatment with DMSO, OSMI-1, or TMG in RAW264.7 cells. GAPDH served as a loading control for **(D–H)**. Data are representative of three independent experiments.

In order to determine the role of the potential FTO O-GlcNAcylation during inflammation, we initially detected whether the FTO protein undergoes OGT-mediated O-GlcNAcylation modification. Co-IP assay revealed that endogenous FTO in BMDMs was associated with OGT and exhibited a positive O-GlcNAc signal when probed with an anti-O-GlcNAc antibody (RL2) (**Figure 1A**). A reverse Co-IP assay further verified this association (**Figure 1B**), suggesting that FTO interacts with the O-GlcNAc transferase OGT.

To further confirm the existence of FTO O-GlcNAcylation, we used metabolic glycan labeling combined with bioorthogonal click chemistry (41, 42). The N-azidoacetylgalactosamine (GalNAz) was incubated with the RAW264.7 cells, in which GalNAz could be converted to N-azidoacetylglucosamine (GlcNAz) through cellular glucose metabolism and used to “tag” glycosylated proteins with azide (**Figure 1C**). Then, GlcNAz activates the hexosamine biosynthesis pathway (HBP) in the presence of uridine triphosphate (UTP) of nucleotide metabolism and generates UDP-GlcNAz, which induces protein O-GlcNAc modification by OGT (32). The cells were lysed, immunoprecipitated with an anti-FTO antibody and protein-A/G magnetic beads, followed by incubation with biotin-dibenzocyclooctyne (DBCO), and then subjected to SDS-PAGE and detection by chemiluminescence using streptavidin conjugated to horseradish peroxidase (HRP). The azide-alkyne cycloaddition of click chemistry demonstrated increased DBCO-biotin binding to O-GlcNAcylated FTO in RAW264.7 cells treated with GalNAz compared to controls without GalNAz (**Figure 1D**).

We also mixed different concentrations of Glucosamine (GlcN) with BMDMs. After incubation for 24 h, the cells were lysed, immunoprecipitated with anti-FTO and protein-A/G magnetic beads, and then subjected to SDS-PAGE and WB analysis using anti-FTO and RL2 antibodies. The results showed increased FTO O-GlcNAcylation when the concentration of GlcN was raised from 5 mM to 20 mM (**Figure 1E**). The exogenous Co-IP experiment also identified the interaction between FTO and OGT (**Figure 1F**). The above results strongly suggest that FTO undergoes O-GlcNAcylation.

Next, we sought to determine whether the O-GlcNAcylation level of FTO depends on OGA and OGT. Co-IP and WB analysis showed that overexpression of OGA decreased the O-GlcNAcylation level of FTO (**Figure 1G**), while overexpression of WT OGT but not its K908A enzyme active site mutant (43) greatly enhanced the O-GlcNAcylation level of Flag-FTO (**Figure 1H**). Moreover, the majority of FTO and OGT colocalized in the cytoplasm in RAW264.7 macrophages (**Figure 1I**). The OGT inhibitor OSMI-1 (44) decreased the cytoplasmic OGT expression as well as the interaction between OGT and FTO as indicated by the decreased merged yellow fluorescence signal in the cytoplasm (**Figure 1I**). In contrast, the OGA inhibitor TMG (45) enhanced the cytoplasmic OGT-FTO interaction (**Figure 1I**). The above data suggest that FTO undergoes OGT-mediated O-GlcNAcylation.

### 3.2 FTO is O-GlcNAcylated at the Ser95 site of the N-terminal region

**Figure 2:**
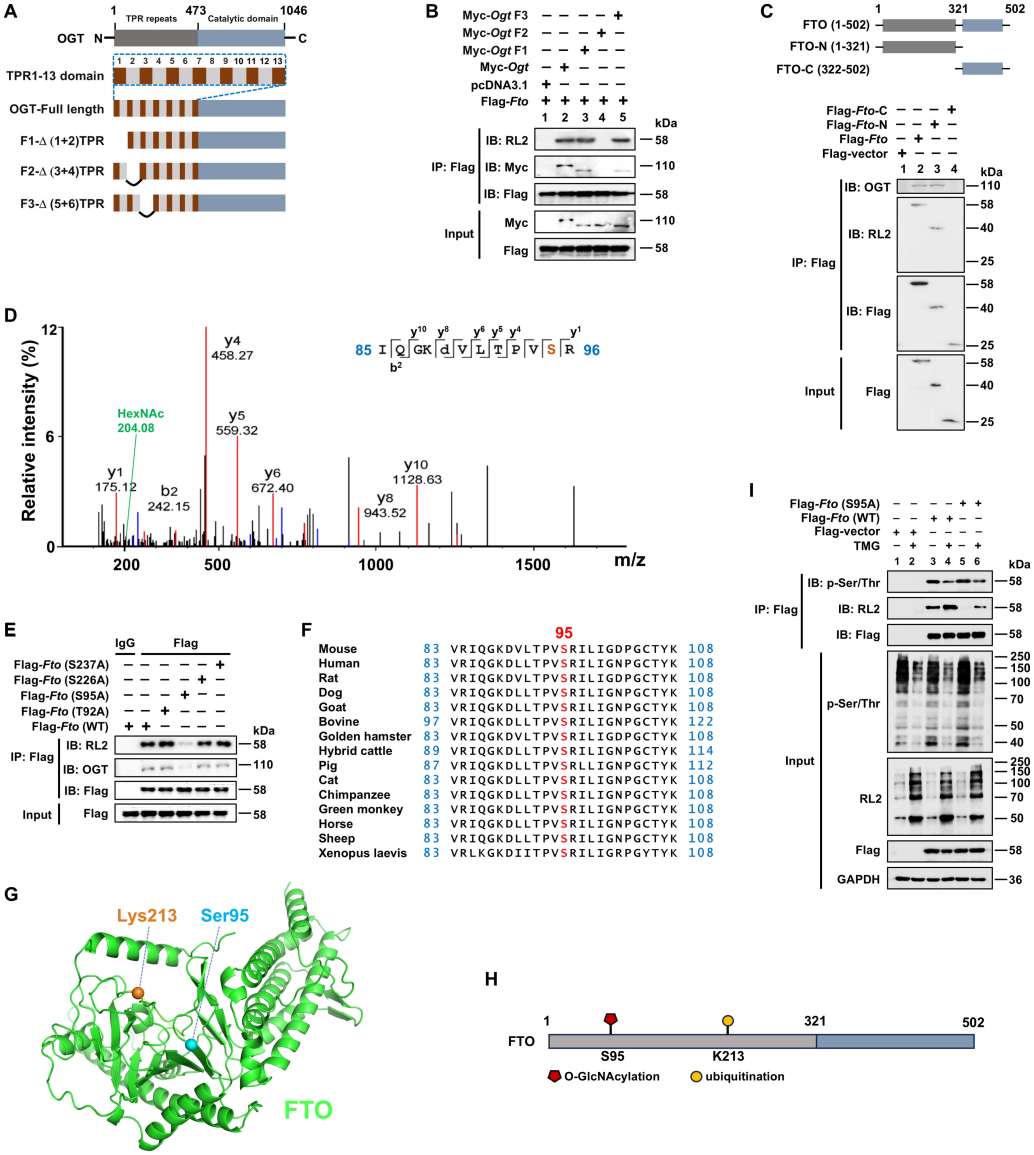
FTO is O-GlcNAcylated at the Ser95 site of the N-terminal region. **(A)** Schematic of full-length and deletion mutants of *Ogt* constructs. **(B)** The HEK293T cells were transfected with different constructs of Myc*-Ogt* and full-length Flag*-Fto* for 48 h, and then subjected to Co-IP with an anti-Flag and immunoblot blot with Abs against RL2, Myc, and Flag, respectively. **(C)** Schematic representation of full-length and deletion mutant constructs of *Fto* (upper panel) and Co-IP (lower panel). The HEK293T cells were transfected with full-length and deletion mutant constructs of Flag*-Fto* for 48 h, and then subjected to Co-IP with an anti-Flag and immunoblot with Abs against OGT, RL2, and Flag, respectively. **(D)** The RAW264.7 cells were transfected with Flag*-Fto* or Flag*-Fto-N* and then subjected to pull-down with an anti-Flag and analysis by LC-MS/MS. **(E)** The HEK293T cells were transfected with Flag*-Fto* or *Fto*-mutants (T92A/S95A/S226A/S237A), and then subjected to Co-IP with an anti-Flag and immunoblot with Abs against OGT, RL2, and Flag, respectively. **(F)** Cross-species conservation analysis of FTO at S95 O-GlcNAcylation site. **(G)** The spatial structure of murine FTO protein with Ser95 (O-GlcNAcylation site) and Lys213 (ubiquitination site) by AlphaFold are shown as blue and orange sticks, respectively. **(H)** The structural domains and PTM sites of FTO protein are indicated. **(I)** Co-IP and immunoblot analysis of the effect of *Fto* (S95A)/TMG on FTO phosphorylation. HEK293T cells were transfected with Flag*-Fto* (WT)/*Fto* (S95A)*/*Flag-vector for 24 h then treated with TMG for 24 h, and subjected to Co-IP with an anti-Flag and immunoblot with Abs against RL2, p-Ser/Thr and Flag, respectively. GAPDH served as a loading control for **(I)**. Data are representative of three independent experiments.

Since OGT is responsible for O-GlcNAcylation, we then determined the key domain for the interaction between OGT and FTO. OGT comprises an N-terminal region containing multiple tetratricopeptide repeat (TPR) units and a C-terminal region consisting of two catalytic domains, of which the TPR domain is responsible for binding the substrate (46). We constructed a Myc-tagged WT *Ogt* and a series of *Ogt* deletion (F1/F2/F3) mutant expression plasmids (**Figure 2A**). After transfection in HEK293T cells, Co-IP and WB analysis showed that the OGT F2 mutant lost its binding with FTO (**Figure 2B**, Lane 4), suggesting that the TPR3-TPR4 region of OGT is needed for its association with FTO. We further constructed FTO N-terminal (FTO-N) and C-terminal (FTO-C) deletion mutants (**Figure 2C**). Using Co-IP and WB analysis, we observed that FTO-C lost binding with OGT (**Figure 2C**, Lane 4), suggesting that the FTO N-terminus binds to the OGT TPR3-TPR4 region.

Furthermore, we identified the precise O-GlcNAcylation site (s) on FTO. Using an anti-Flag-FTO antibody pulldown from Flag-tagged full-length FTO or N-terminal fragment FTO expression plasmids-transfected RAW264.7 cells, subsequently for SDS-PAGE, and LC-MS/MS analysis, we found that FTO O-GlcNAcylation mainly occurred on a peptide (IQGKDVLT_92_PVS_95_R) that contains two potential O-GlcNAcylation sites (Thr92 and Ser95) with the prominent N-acetylhexosamine (HexNAc) ion peak at 204.08 m/z (**Figure 2D**), which is a characteristic marker of O-GlcNAcylation. This peak indicates that FTO undergoes O-GlcNAcylation at either of these two sites, as the addition of HexNAc is a hallmark of this post-translational modification. To further determine the specific potential FTO O-GlcNAcylation modification site (s), two single mutant constructs Flag-*Fto* (T92A)/(S95A) were generated and transfected into HEK293T cells, respectively. Co-IP and WB showed that the S95A mutant, but not the T92A mutant, showed a dramatically diminished level of FTO O-GlcNAcylation, as well as the impaired interaction between FTO and OGT (**Figure 2E**), validating that Ser95 is the O-GlcNAcylation site of FTO.

In addition, another two potential O-GlcNAcylation sites Ser226 and Ser237 (predicted by two different online databases: YinOYang 1.2 Server and OGTSite) were also mutated as Ala, respectively, and examined as well. We found these two mutations did not affect the O-GlcNAcylation levels of FTO and FTO-OGT interaction (**Figure 2E**).

Ser95 is located at the PVT/S (proline–valine–threonine/serine) motif, which has been reported to be conserved in approximately half of the O-GlcNAcylated proteins identified to date (47). Additionally, FTO Ser95 is well conserved among metazoan species (**Figure 2F**), indicating that it serves an evolutionarily conserved role in regulating the FTO protein. Thus, our above results first demonstrated that Ser95 is the critical O-GlcNAcylation site of FTO (**Figures 2G, H**).

Both phosphorylation and O-GlcNAcylation can modify Ser and Thr residues, suggesting dynamic mutual regulation between O-GlcNAcylation and phosphorylation (47). We next examined whether O-GlcNAcylation of FTO at Ser95 could affect its phosphorylation. We found that the FTO Ser95A mutant did not affect FTO p-Ser/Thr levels (**Figure 2I**, Lane 5 *vs.* 3). However, the OGA inhibitor TMG treatment promoted the total O-GlcNAcylation levels of FTO and inhibited FTO phosphorylation (**Figure 2I**, Lane 4 *vs.* 3). These results suggest that promotion of total O-GlcNAcylation modification by the OGA inhibitor can suppress FTO phosphorylation level, while mutation of FTO O-GlcNAcylation at the Ser95 site does not affect FTO phosphorylation level.

### 3.3 LPS enhances FTO O-GlcNAcylation relying on FOXO1-regulated GFAT2 expression in macrophages

**Figure 3:**
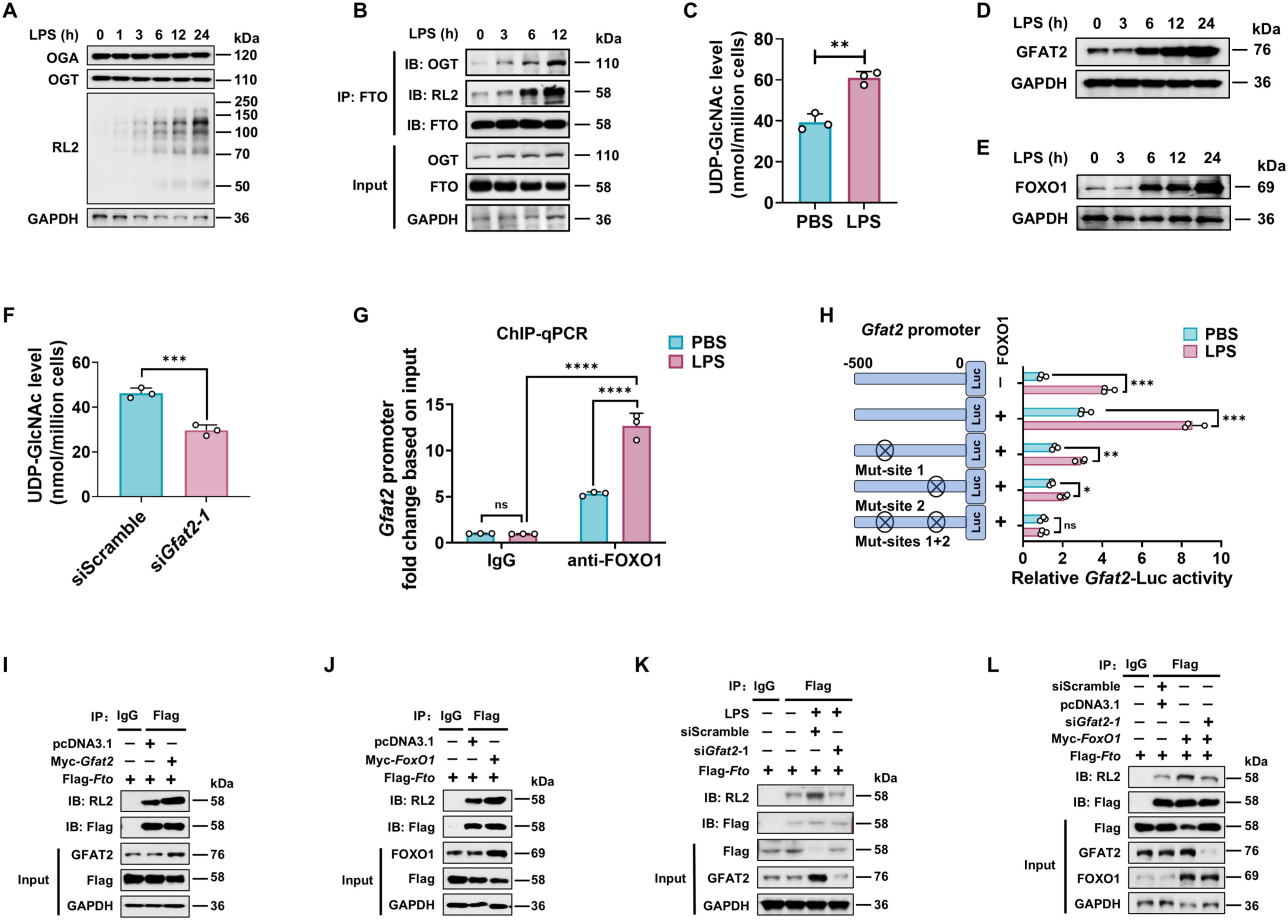
LPS enhances FTO O-GlcNAcylation relying on GFAT2 in macrophages. **(A)** Immunoblot of OGA/OGT protein expression, and total cellular proteins O-GlcNAcylation levels in LPS treated BMDMs. **(B)** Analysis of the effect of LPS on FTO O-GlcNAcylation and FTO-OGT interaction in BMDMs. BMDMs were treated with LPS for the indicated time and subjected to Co-IP with an anti-FTO and immunoblot with Abs against OGT, RL2, and FTO, respectively. **(C)** HPLC-MS analysis of UDP-GlcNAc content from BMDMs treated with PBS or LPS. **(D, E)** Immunoblot was used to assess the expression of GFAT2 **(D)** and FOXO1 **(E)** in BMDMs treated with LPS for the indicated time. **(F)** BMDMs were transfected with si*Gfat2–*1 or siScramble for 48 h, and then subjected to UDP-GlcNAc content analysis by HPLC-MS. **(G)** ChIP-qPCR assay of the binding of FOXO1 protein to the promoter region of *Gfat2* in BMDMs treated with LPS or PBS for 12 (h) Rabbit IgG served as the negative control for immunoprecipitation. **(H)** Dual luciferase reporter assay of the activity of the *Gfat2* promoter. RAW264.7 cells were co-transfected with *Gfat2*-luciferase reporter construct, a Renilla luciferase expression plasmid, and either pcDNA3.1 or pcDNA3.1-*FoxO1* and then stimulated with or without LPS for 12 (h) **(I-L)** Co-IP and immunoblot analysis of the effects of *Gfat2* **(I, K, L)** and *FoxO1* **(J, L)** silencing **(K, L)** or overexpression **(I, J, L)**, or *FoxO1* overexpression plus *Gfat2* silencing **(L)** on FTO O-GlcNAcylation. RAW264.7 cells were transfected with Flag-*Fto* and Myc-*Gfat2/*Myc-*FoxO1*/si*Gfat2–*1 for 48 h and then stimulated with or without LPS. The cells were subjected to Co-IP with an anti-Flag and immunoblot with Abs against GFAT2, FOXO1, RL2, and Flag, respectively. GAPDH served as a loading control for **(A, B)**, **(D, E)**, and **(I–L)**. Data are representative of three independent experiments. Data represent the mean ± SD with significance determined by two-tailed unpaired Student’s t-test for **(C, F, H)**, and by two-way ANOVA followed by Tukey’s multiple comparison tests for **(G)**. ns, not significant; *p<0.05; **p<0.01; ***p<0.001; ****p<0.0001.

We next assessed the level of FTO O-GlcNAcylation in macrophages at a series of time points after LPS stimulation. We observed that LPS-stimulated BMDMs enhanced total protein O-GlcNAcylation, but did not exhibit any detectable change in the protein expression levels of OGT and OGA (**Figure 3A**). OGT showed a weak association with FTO in unstimulated macrophages, but this association, as well as O-GlcNAcylated FTO, increased upon LPS stimulation (**Figure 3B**). This result suggests that LPS does not affect the protein expression levels of OGT and OGA, while LPS increases FTO O-GlcNAcylation by promoting the interaction between OGT and FTO.

Since LPS-enhanced FTO O-GlcNAcylation does not depend on both OGT and OGA protein expression (**Figure 3A**), we measured UDP-GlcNAc levels after LPS stimulation using HPLC-MS. Consistent with increased total protein O-GlcNAcylation, UDP-GlcNAc levels rose following LPS stimulation (**Figure 3C**), indicating that LPS amplified HBP activity and increased UDP-GlcNAc incorporation of FTO.

Glutamine fructose-6-phosphate amidotransferase 2 (GFAT2) is a critical enzyme that catalyzes the rate-limiting step of the HBP, and forkhead box O1 (FOXO1) has been reported as a transcription factor of the *Gfat2* gene (48). We also observed that LPS treatment increased both mRNA (**Supplementary Figures 1A, B**) and protein (**Figures 3D, E**) expression of the GFAT2 and FOXO1 in a time-dependent manner in macrophages. In addition, silence of *Gfat2* attenuated the UDP-GlcNAc abundance of macrophages (**Figure 3F**; **Supplementary Figure 1C**).

Next, ChIP-qPCR and dual luciferase reporter assay were used to determine whether FOXO1 binds to the *Gfat2* promoter during LPS stimulation. The result revealed an increase in FOXO1 binding to the *Gfat2* promoter in the LPS-treated macrophages compared with the control groups (PBS and isotype IgG) (**Figure 3G**). Mouse *Gfat2* promoter contains two FOXO1 recognition sites (referred to as site 1 and site 2) (48). We constructed four luciferase reporter gene plasmids including the *Gfat*2 promoter region (containing the -500-bp region), each one or both FOXO1 binding sites mutation of the *Gfat*2 promoter. After LPS or PBS stimulation, we measured the luciferase activities of the promoter in RAW264.7 cells. The results revealed that the activity of the *Gfat*2 promoter-reporter gene was markedly increased by co-transfection with FOXO1 after LPS stimulation (**Figure 3H**). The LPS effect was markedly reduced by the site 1/2 mutation of the *Gfat2* promoter and abolished when both sites were mutated (**Figure 3H**), suggesting that these two sites are important for the regulation of *Gfat2* expression by FOXO1. LPS stimulation increases the FOXO1-regulated GFAT2 expression in macrophages.

Next, we examined the effect of GFAT2 or FOXO1 on the FTO O-GlcNAcylation. Co-IP and WB analysis showed that overexpression of *Gfat2* or *FoxO1* promoted FTO O-GlcNAcylation (**Figures 3I, J**). As expected, either *Gfat2* or *FoxO1* knockdown decreased FTO O-GlcNAcylation (**Supplementary Figures 1C–F**). LPS-increased FTO O-GlcNAcylation was abolished following *Gfat2* knockdown (**Figure 3K**). *Gfat2* knockdown reversed the FOXO1-induced increase in FTO O-GlcNAcylation (**Figure 3L**).

The above results demonstrate that LPS does not affect the protein expression levels of OGT and OGA, However, LPS stimulation enhances GFAT2 and FOXO1 proteins expression. FTO O-GlcNAcylation relying on FOXO1-regulated GFAT2 expression.

### 3.4 O-GlcNAcylation of FTO can promote the attachment of K48-linked ubiquitin chains to the FTO

**Figure 4:**
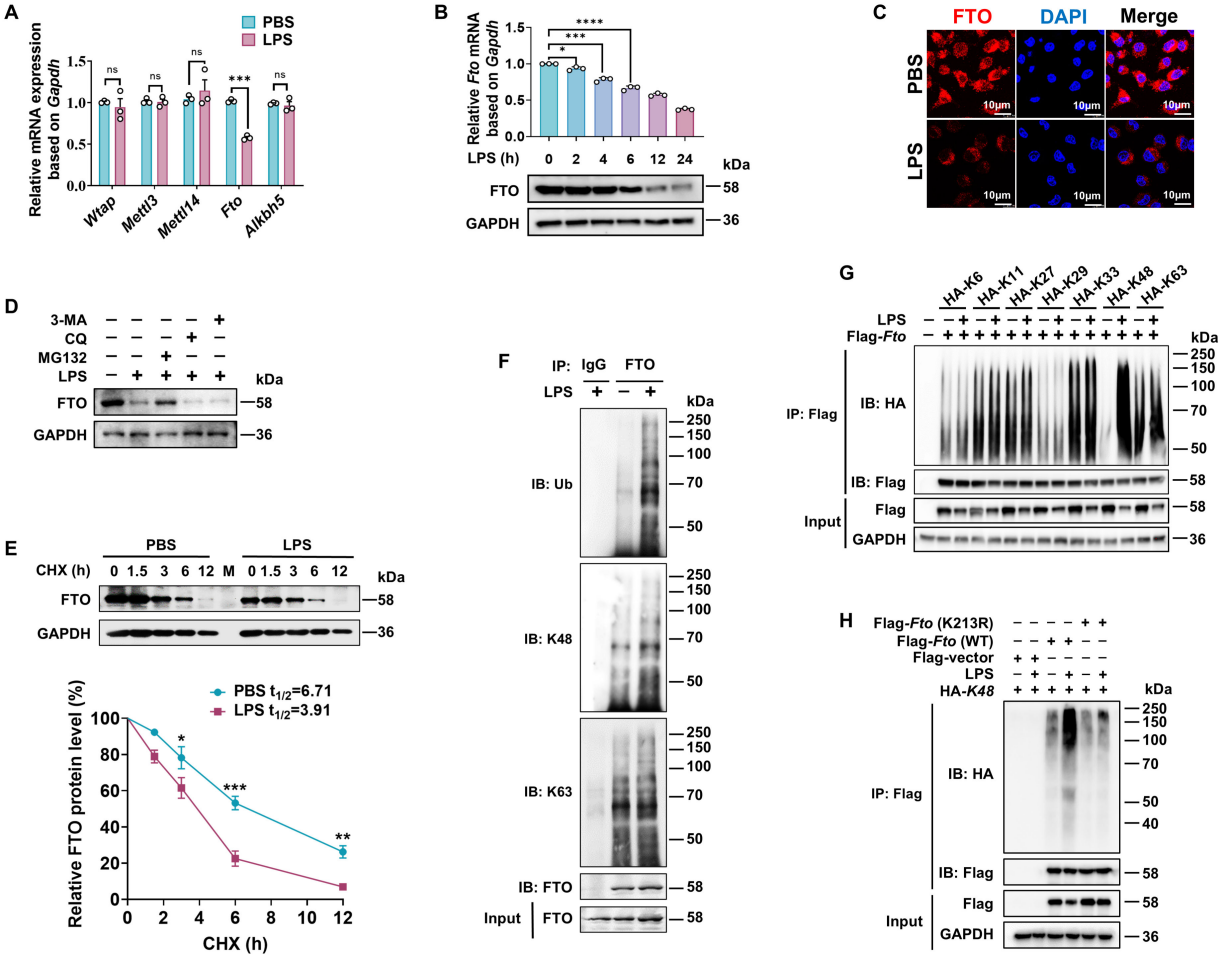
LPS promotes K48 ubiquitination degradation of FTO in macrophages. **(A)** RT-qPCR analysis of *Wtap, Mettl3, Mettl14, Fto*, and *Alkbh5* expression in BMDMs induced by LPS. **(B)** RT-qPCR and immunoblot analysis of FTO expression in BMDMs treated with LPS for the indicated time. **(C)** Immunofluorescence of FTO in RAW264.7 cells treated with PBS or LPS for 12 (h) **(D)** Immunoblot analysis of FTO in BMDMs incubated with MG132, CQ, 3-MA, or DMSO for 24 h, followed by treatment with LPS or PBS for 12 (h) **(E)** Immunoblot and quantitative analysis of FTO in BMDMs incubated with PBS or LPS for 12 h, then treated with CHX for the indicated time. **(F)** BMDMs were treated with LPS or PBS for 12 h and then subjected to Co-IP with an anti-FTO and immunoblot with Abs against K48, K63, Ub, and FTO, respectively. **(G)** RAW264.7 cells were transfected with the indicated plasmids (HA-K6/K11/K27/K33/K29/K48/K63) for 48 h and then treated with LPS or PBS for 12 (h) The cell lysates were subjected to Co-IP with an anti-Flag and immunoblot with Abs against Flag and HA. **(H)** Co-IP and immunoblot analysis of FTO (WT) and FTO (K213R) ubiquitination in RAW264.7 cells. GAPDH served as a loading control for **(B)**, **(D, E)**, and **(G, H)**. Data are representative of three independent experiments. Data represent the mean ± SD with significance determined by two-tailed unpaired Student’s t-test for **(A, E)**, and by one-way ANOVA followed by Dunnett multiple comparisons tests for **(B)**. ns, not significant; *p<0.05; **p<0.01; ***p<0.001; ****p<0.0001.

Since m^6^A is installed by the m^6^A methyltransferase complex (m^6^A writers), which includes complex methyltransferase-like 3 (METTL3), METTL14, and Wilms’ tumor-1-associating protein (WTAP), while removed by m^6^A erasers such as FTO and AlkB homolog 5 (ALKBH5) (49), we quantified the expression patterns of m^6^A writers (*Wtap*, *Mettl3*, and *Mettl14*) and erasers (*Fto* and *Alkbh5*) in mouse BMDMs under LPS stimulation by RT-qPCR. The level of *Fto* mRNA was significantly downregulated, whereas other components displayed no significant changes (**Figure 4A**). Moreover, the level of FTO was downregulated by LPS, evidenced by reductions at both mRNA and protein levels in a time-dependent manner (**Figure 4B**). Immunofluorescence assay also indicated diminished FTO protein expression and intensity in RAW264.7 macrophages after LPS treatment. (**Figure 4C**).

A previous report has elucidated that LPS-induced downregulation of FTO at both transcriptional and translational levels (22). Nevertheless, it remains to be further verified whether LPS also affects FTO expression at the level of posttranslational modification. We observed that the proteasomal inhibitor MG132 at a concentration of 100 nM prevented about 60% reduction of FTO protein expression level induced by LPS, while the lysosome inhibitor CQ or the autophagy inhibitor 3-MA at a concentration of 20 μM or 5 mM did not affect the protein expression levels of FTO (**Figure 4D**), suggesting that LPS induced FTO proteasome degradation. The protein synthesis inhibitor CHX has been widely used to measure the half-lives of proteins because it can block the biosynthesis of proteins non-selectively. CHX chase assay demonstrated that LPS accelerates FTO protein degradation (**Figure 4E**), suggesting that LPS decreases FTO expression at the level of posttranslational level.

Next, we found that LPS stimulation induced further prominent enhancement in total and K48-linked ubiquitination of FTO but had a minor effect on K63-linked ubiquitination (**Figure 4F**). There are seven K residues in the ubiquitin chain (K6, K11, K27, K29, K33, K48, and K63), which can form polyubiquitin chains. HEK293T cells were co-transfected with Flag-*Fto* and ubiquitin substitution mutants, where one of the seven lysine residues was retained as lysine, and the others were replaced with arginine. Notably, LPS stimulation prominently enhanced the K48-linked polyubiquitination of FTO but had a minor effect on the FTO ubiquitination of other types of linkages (**Figure 4G**). It was reported that FTO undergoes active ubiquitination on the evolutionarily conserved Lys213 (Lys216 in humans) residue (50). We also found that LPS-induced FTO K48 ubiquitination is dependent on the K213 site, as the *Fto* (K213R) mutant showed dramatically decreased ubiquitination compared with WT *Fto* upon LPS stimulation (**Figure 4H**). Together, these results demonstrate that LPS leads to the accelerated K48-ubiquitin-proteasome-mediated degradation of FTO at K213 in macrophages.

**Figure 5:**
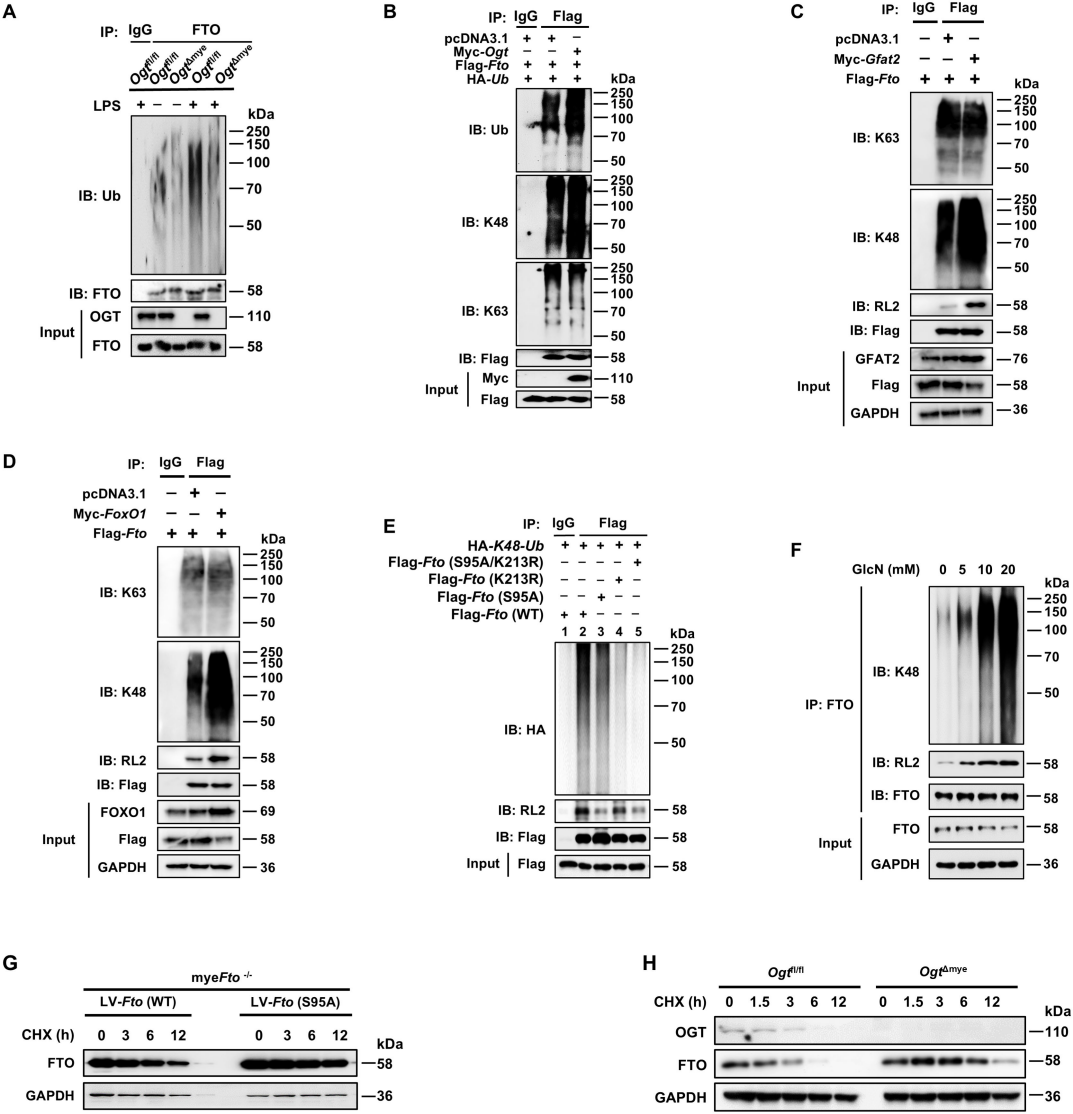
O-GlcNAcylation of FTO promotes its K48-ubiquitination degradation and decreases FTO stability. **(A)** Co-IP and immunoblot analysis of FTO ubiquitination in *Ogt* ^fl/fl^ and *Ogt* ^Δmye^ BMDMs treated with LPS or PBS for 12 (h) **(B)** Co-IP and immunoblot analysis of the effect of OGT on FTO ubiquitination with the indicated Abs. HEK293T cells were transfected with Flag*-Fto*, pcDNA3.1, Myc*-Ogt*, and HA*-Ub* for 48 h and subjected to Co-IP with an anti-Flag and immunoblot with Abs against Ub, K48, K63, Myc, and Flag, respectively. **(C)** Co-IP and immunoblot analysis of the effect of GFAT2 on FTO ubiquitination with the indicated Abs. HEK293T cells were transfected with Flag*-Fto*, pcDNA3.1, and Myc*-Gfat2* for 48 h and subjected to Co-IP with an anti-Flag and immunoblot with Abs against K48, K63, RL2, GFAT2, and Flag, respectively. **(D)** Co-IP and immunoblot analysis of the effect of FOXO1 on FTO ubiquitination with the indicated Abs. HEK293T cells were transfected with Flag*-Fto*, pcDNA3.1, and Myc*-FoxO1* for 48 h and subjected to Co-IP with an anti-Flag and immunoblot with Abs against K48, K63, RL2, FoxO1, and Flag, respectively. **(E)** The HEK293T cells were co-transfected with Flag*-Fto* (WT/S95A/K213R) or HA*-K48-Ub* plasmids for 48 h and then subjected to Co-IP with an anti-Flag and immunoblot with Abs against HA, RL2, and Flag. **(F)** BMDMs were incubated with the indicated concentrations of GlcN for 24 h, and then subjected to Co-IP with an anti-FTO and immunoblot with Abs against K48, RL2, and FTO, respectively. **(G)** The mye*Fto* ^-/-^ BMDMs were infected with LV-*Fto* (WT) or *Fto* (S95A) and treated with CHX for the indicated time. Immunoblot analysis of FTO expression in mye*Fto* ^-/-^ BMDMs. **(H)** Immunoblot of FTO expression in *Ogt* ^fl/fl^ and *Ogt* ^Δmye^ BMDMs treated with CHX for the indicated time. GAPDH served as a loading control for **(C, D)** and **(F–H)**. Data are representative of three independent experiments.

We sought to determine whether FTO O-GlcNAcylation could affect the ubiquitination-dependent degradation of FTO. We generated mice with a myeloid-specific *Ogt* deletion (*Ogt* ^Δmye^), as global *Ogt* ablation is lethal (51). LPS induced increased ubiquitination of FTO in *Ogt* ^fl/fl^ BMDMs (**Figure 5A**). However, this increased FTO ubiquitination did not occur in *Ogt* ^Δmye^ BMDMs (**Figure 5A**). Overexpression of OGT induced enhancement in total and K48-ubiquitination of FTO but did not affect K63-ubiquitination (**Figure 5B**). Moreover, overexpression of GFAT2 or FOXO1 also increased K48-ubiquitination of FTO and decreased the protein expression of FTO, but did not affect K63-ubiquitination (**Figures 5C, D**). We also observed that the FTO O-GlcNAcylation mutant at Ser95 decreased K48 ubiquitination and O-GlcNAcylation (**Figure 5E**, Lane 3), suggesting that FTO ubiquitination depends on FTO O-GlcNAcylation at Ser95 site. The FTO ubiquitination mutant *Fto* (K213R) or *Fto* (K213R/S95A) double mutation prominently reduced the K48 ubiquitination of FTO, but *Fto* (K213R) did not affect FTO O-GlcNAcylation (**Figure 5E**, Lane 4), suggesting that Lys213 ubiquitination sequentially occurs after Ser95 O-GlcNAcylation of FTO, and Lys213 ubiquitination of FTO does not affect its O-GlcNAcylation. As expected, administration of GlcN markedly increased FTO O-GlcNAcylation and K48 ubiquitination in BMDMs in a GlcN dose-dependent manner (**Figure 5F**). The above results suggest that FTO Ser 95 O-GlcNAcylation facilitates FTO K48-ubiquitination at K213, but Lys213 ubiquitination of FTO does not affect FTO O-GlcNAcylation.

### 3.5 FTO O-GlcNAcylation at the S95 site decreases the stability of FTO protein

To investigate the effect of FTO O-GlcNAcylation on its stability, the BMDMs were isolated from mye*Fto* ^-/-^ mice, infected with LV-*Fto* (S95A) or LV-*Fto* (WT), and then treated with CHX. WB showed that the FTO S95A mutant protein, but not the WT FTO protein, displayed a longer half-life (**Figure 5G**), suggesting that the FTO O-GlcNAcylation at Ser95 destabilizes FTO, and deficiency of FTO O-GlcNAcylation at Ser95 facilitates FTO stability. CHX chase analysis also showed that FTO degradation was decreased in *Ogt* knockout (*Ogt* ^Δmye^) BMDMs compared with those in *Ogt* ^fl/fl^ BMDMs (**Figure 5H**). These suggest that OGT-mediated O-GlcNAcylation of FTO at the S95 site decreases its stability.

Since FTO O-GlcNAcylation decreases its stability, we next assessed whether OGT overexpression could suppress FTO-mediated m^6^A demethylation levels. Accordingly, global RNA m^6^A demethylation modification levels increased after *Fto* overexpression **(Supplementary Figure 2A**, Column 2 *vs.* 1), but decreased in RAW264.7 cells overexpressing both *Fto* and *Ogt* (**Supplementary Figure 2A**, Column 3 *vs.* 2), suggesting that *Ogt* overexpression decreases FTO-mediated m^6^A demethylation. In addition, the protein levels of FTO were decreased following the upregulation of O-GlcNAcylation with TMG (**Supplementary Figure 2B**) and increased following the downregulation of O-GlcNAcylation with OSMI-1 (**Supplementary Figure 2C**) in a dose-dependent manner, which profoundly suggests that FTO O-GlcNAcylation decreases the stability of FTO protein and downregulates the protein levels of FTO.

### 3.6 FTO O-GlcNAcylation at S95 site recruits E3 ubiquitin ligase TRIM21 to promote FTO K48-ubiquitination degradation

**Figure 6:**
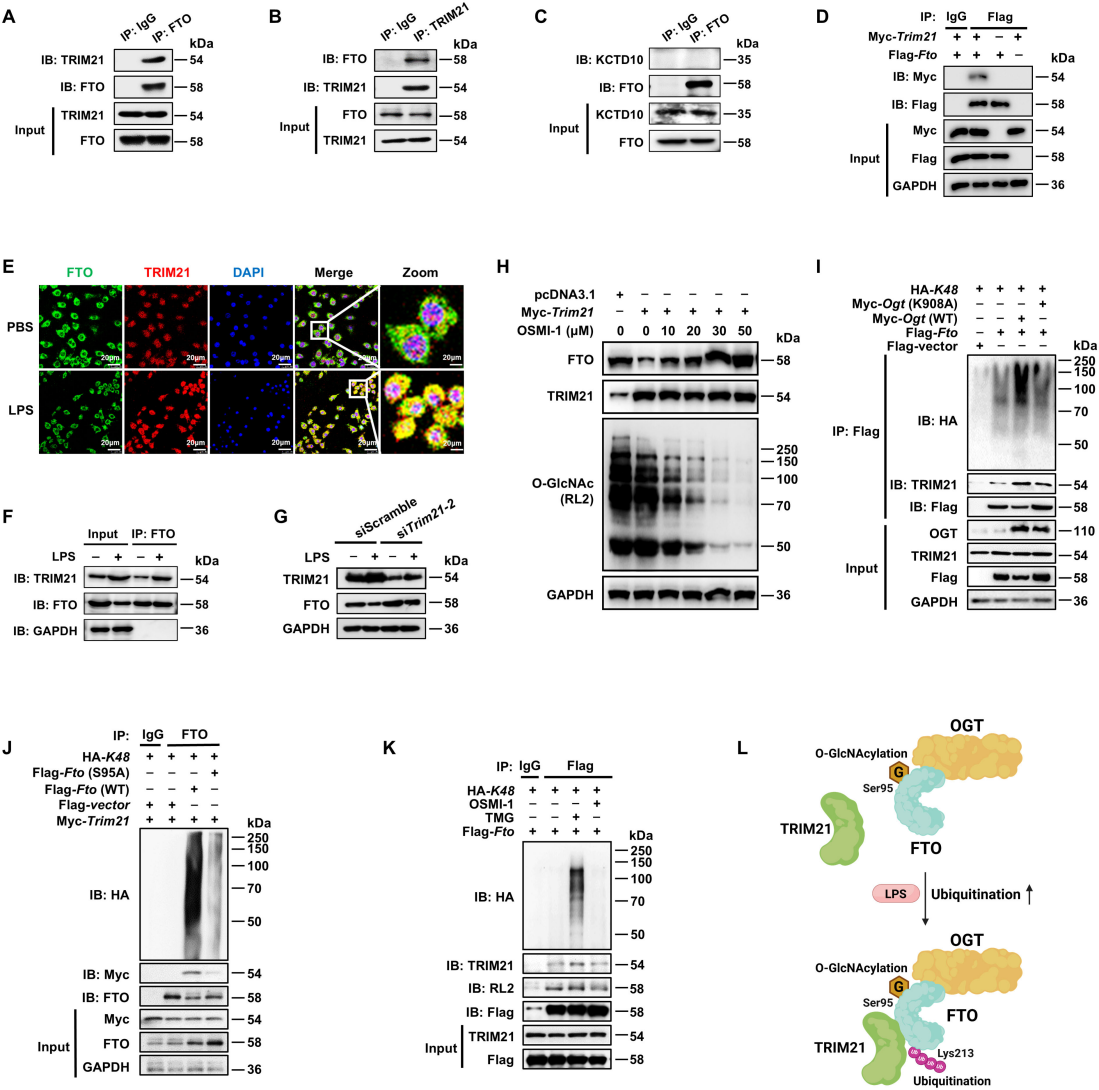
FTO O-GlcNAcylation recruits E3 ubiquitin ligase TRIM21 to promote FTO K48-ubiquitination. **(A, B)** The cell lysates of BMDMs were subjected to Co-IP with anti-FTO **(A)** or anti-TRIM21 **(B)** and immunoblot with Abs against TRIM21 and FTO. **(C)** Co-IP and immunoblot analysis of FTO binding with KCTD10. Total FTO from BMDMs was Co-IP with anti-FTO Ab, followed by immunoblotting with anti-FTO and anti-KCTD10 Abs. **(D)** Co-IP and immunoblot analysis of the interaction between exogenous FTO and TRIM21. HEK293T cells were transfected with Flag*-Fto* and Myc-*Trim21* for 48 h and subjected to Co-IP with an anti-Flag and immunoblot with Abs against Myc and Flag, respectively. **(E)** Immunofluorescence analysis of colocalization of FTO (Alexa Fluor 488) and TRIM21 (Alexa Fluor 594) in RAW264.7 cells with or without LPS stimulation for 24 (h) DAPI was used to identify the nucleus. **(F)** Co-IP and immunoblot analysis of FTO binding with TRIM21 in the presence or absence of LPS stimulation. BMDMs were exposed to PBS or LPS for 12 h, and then cell lysates were subjected to Co-IP with an anti-FTO and immunoblot with Abs against FTO and TRIM21. **(G)** Immunoblot analysis of FTO and TRIM21 expression in RAW264.7 after *Trim21* silencing. **(H)** Immunoblot analysis of FTO expression. RAW264.7 cells were transfected with *Trim21* for 48 h and then treated with OSMI-1 for 24 (h) **(I)** The HEK293T cells were co-transfected with Flag*-Fto*/Flag*-vector*, HA*-K48*, and Myc*-Ogt* (WT)/*Ogt* (K908A) for 48 h, and then subjected to Co-IP and immunoblot for analysis of the effects of OGT on FTO-TRIM21 binding and FTO ubiquitination. **(J)** The HEK293T cells were Co-transfected with Flag*-Fto* (WT)/*Fto* (S95A)*/*Flag-vector, HA*-K48*, and Myc*-Trim21* for 48 h, and then subjected to Co-IP and immunoblot for analysis of the interaction between FTO and TRIM21 and FTO ubiquitination. **(K)** RAW264.7 cells were transfected with Flag*-Fto* and HA*-K48* for 48 h, then treated with TMG or OSMI-1 for 24 h, and subjected to Co-IP and immunoblot analysis. **(L)** The working model of FTO O-GlcNAcylation at Ser95 site recruiting TRIM21 to promote FTO K48-ubiquitination. GAPDH served as a loading control for **(D)** and **(F–J)**. Data are representative of three independent experiments. .

The ubiquitin-proteasome system is mainly composed of the ubiquitin-activating enzyme (E1), ubiquitin-crosslinking enzyme (E2), ubiquitin ligase (E3) and the 26S proteasome (52–54). Among these components, the E3 ubiquitin ligase is responsible for target protein recognition and mediates the degradation of substrates (55, 56). We performed IP-MS analysis by using anti-FTO and protein protein-A/G magnetic beads from lysates of BMDMs, and identified two E3 ligases in association with FTO (**Supplementary Table 3**): the tripartite motif family member TRIM21 (also known as Ro52) and BTB/POZ domain-containing adapter for CUL3-mediated RhoA degradation protein (KCTD10). Then we confirmed the binding between TRIM21 and FTO by Co-IP with anti-FTO and WB analysis (**Figure 6A**), with further support from reverse Co-IP findings with anti-TRIM21 (**Figure 6B**). However, there was no interaction between KCTD10 and FTO by IP and WB with anti-KCTD10 (**Figure 6C**). The exogenous Co-IP experiment also identified the interaction between FTO and TRIM21 (**Figure 6D**).

TRIM21 consists of an N-terminal RING domain, B-box domain, central coiled-coil domain, and terminal PRY/SPRY domain, among which the RING domain is mainly responsible for its E3 ligase activity (57). We found that full-length *Trim21* enhanced the total (**Supplementary Figure 3A**) and K48-linked (**Supplementary Figure 3B**) ubiquitination of FTO, but *Trim21*(δRING), the RING structure deletion mutant of TRIM21, did not affect the total and K48-ubiquitination of FTO (**Supplementary Figures 3A, B**). Moreover, either full-length or *Trim21*(δRING) did not affect the K63-ubiquitination of FTO (**Supplementary Figure 3C**). Further, we determined that LPS treatment increased the colocalization of FTO and TRIM21 in RAW264.7 cells, indicated by an enhanced fluorescence signal (**Figure 6E**). Co-IP and WB assays also showed that LPS increased the expression of TRIM21 as well as the interaction between FTO and TRIM21 in BMDMs (**Figure 6F**). We also observed that *Trim21* knockdown mitigated the LPS-induced reduction of FTO (**Figure 6G**, **Supplementary Figure 3D**). These results strongly demonstrate that LPS promotes TRIM21-mediated K48-ubiquitination degradation of FTO.

We found that OGT inhibitor OSMI-1 induced lower FTO O-GlcNAcylation and further reversed FTO degradation after TRIM21 overexpression (**Figure 6H**). As expected, *Ogt* (WT) rather than *Ogt* (K908A) mutant induced enhancement of interaction between FTO and TRIM21 as well as FTO ubiquitination (**Figure 6I**). Moreover, compared to wild-type FTO, the S95A mutant lost its association with TRIM21 (**Figure 6J**). The OGA inhibitor TMG resulted in FTO O-GlcNAcylation, enhanced the interaction between FTO and TRIM21, and thus elevated K48-ubiquitination of FTO, but the opposite phenomenon was observed in cells pretreated with the OGT inhibitor OSMI-1 (**Figure 6K**). The above results strongly demonstrate that OGT-mediated FTO O-GlcNAcylation facilitates the interaction between FTO and TRIM21 to promote FTO K48-ubiquitination (**Figure 6L**).

### 3.7 FTO O-GlcNAcylation mutation at Ser95 exacerbates *S. Typhimurium* or LPS-induced sepsis in mice

To clarify the role of FTO in the inflammatory response, both mye*Fto* **^-/-^** and parental *Fto* ^fl/fl^ mouse BMDMs were stimulated with LPS. We found that the inflammatory cytokines TNF-α/IL-1β/IL-6 levels were decreased in mye*Fto* **^-/-^** BMDMs compared with those in *Fto* ^fl/fl^ BMDMs (**Supplementary Figure 4A**). Conversely, exogenous lentivirus LV-*Fto* infection of wild-type BMDMs followed by LPS stimulation increased TNF-α/IL-1β/IL-6 cytokine secretion compared to the LV-Ctrl group (**Supplementary Figure 4B** **).** Moreover, the rescue of *Fto* in mye*Fto* ^-/-^ BMDMs by LV-*Fto* also led to increased secretion of these cytokines during LPS stimulation (**Supplementary Figure 4C**).

We further examined the inflammatory response in mye*Fto* **^-/-^** and parental *Fto* ^fl/fl^ mice using the LPS-induced sepsis model (**Supplementary Figure 4D**). Following LPS injection, much higher survival rates and lower levels of serum TNF-α/IL-1β/IL-6 cytokines were detected in the mye*Fto* ^-/-^ mice compared to those in the *Fto* ^fl/fl^ counterparts (**Supplementary Figures 4E, F** **).** The above data suggest that myeloid-specific *Fto* deletion suppresses LPS-induced macrophage inflammatory response and septic mouse mortality, while FTO promotes macrophage inflammation during LPS stimulation.

**Figure 7:**
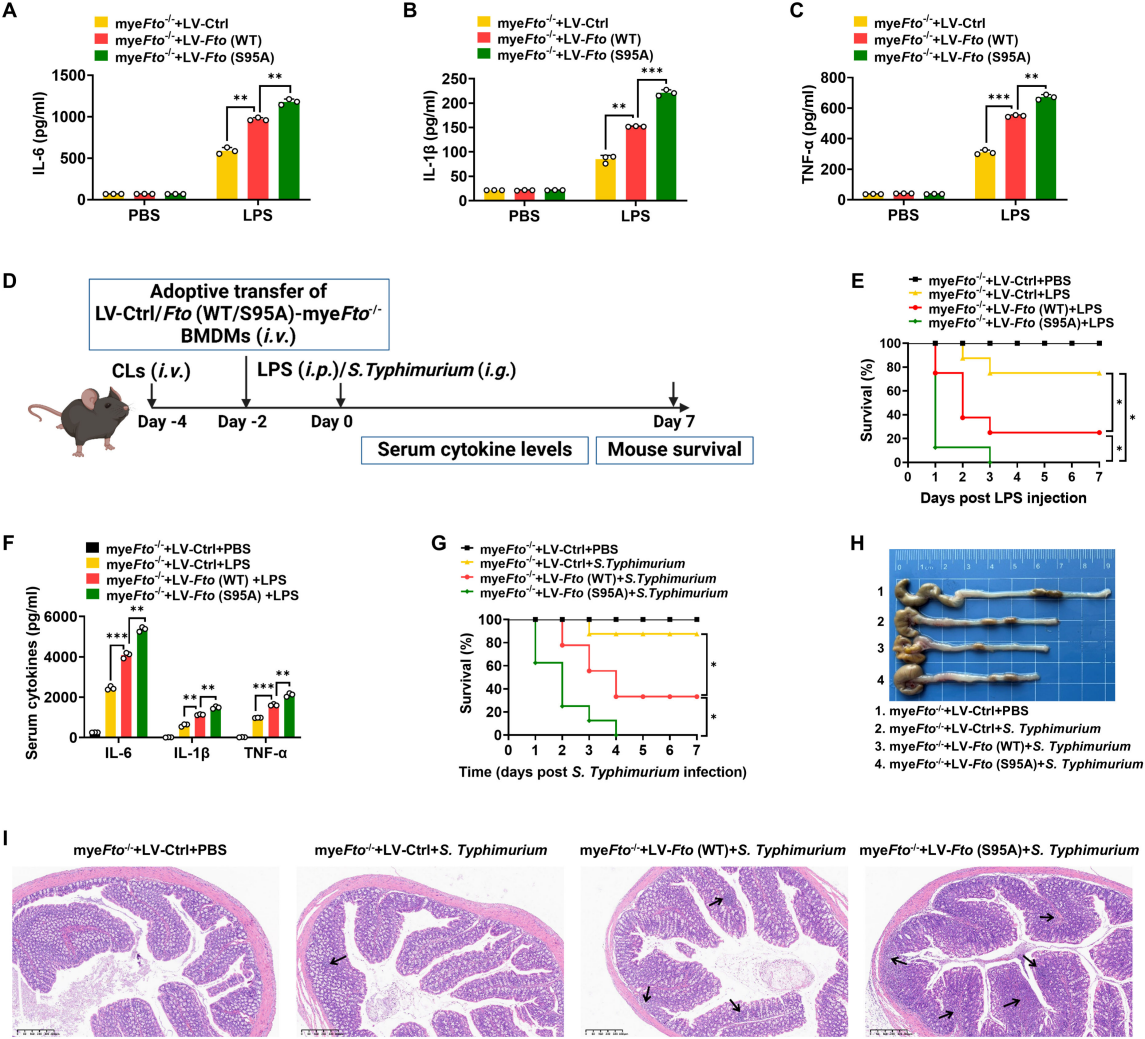
FTO O-GlcNAcylation mutation at Ser95 exacerbates inflammatory response and *S. Typhimurium* or LPS-induced sepsis in mice. **(A–C)** The mye*Fto* ^-/-^ BMDMs were infected with LV-Ctrl/*Fto* (WT)/(S95A) for 48 h following PBS or LPS stimulation for 12 h. The supernatant IL-6/IL-1β/TNF-α concentrations of BMDMs were assessed by ELISA. **(D)** The illustration of macrophage depletion and reconstitution procedure. CLs: clodronate liposomes. **(E)** Kaplan–Meier survival curves of macrophage-depleted mice reconstituted with LV-Ctrl/*Fto* (WT)/(S95A)-infected mye*Fto* ^-/-^ BMDMs followed by PBS or LPS (20 mg/kg, mouse) challenge. **(F)** ELISA of serum IL-6/IL-1β/TNF-α concentrations from mice **(E)** injected with PBS or LPS (20 mg/kg, mouse) for 24 h. **(G)** Kaplan–Meier survival curves of macrophage-depleted mice reconstituted with LV-Ctrl/*Fto* (WT)/(S95A)-infected mye*Fto* ^-/-^ BMDMs followed by *S. Typhimurium* infection. **(H)** Macroscopic picture of the colon between each group of mice as indicated. n = 3 mice/group. **(I)** HE staining of colon tissues from each group of mice. n = 3 mice/group. Log-rank (Mantel-Cox) test was used to assess the statistical difference for **(E, G)**. 8 mice were in each group for **(E, G)**. Data are representative of three independent experiments. Data represent the mean ± SD with significance determined by one-way ANOVA followed by Tukey’s multiple comparison tests for **(A–C)** and **(F)**. *p<0.05; **p<0.01; ***p<0.001.

Since FTO O-GlcNAcylation-induced FTO ubiquitination degradation, we next determined the effects of FTO O-GlcNAcylation at Ser95 on LPS-induced inflammatory cytokines in macrophages. As expected, we found that *Fto* (S95A)-rescued mye*Fto* ^-/-^ BMDMs showed increased IL-1β/IL-6/TNF-α cytokines production compared with *Fto* (WT)-rescued mye*Fto* ^-/-^ BMDMs under LPS stimulation (**Figures 7A–C**). These results suggest that FTO O-GlcNAcylation mutation at Ser95 exacerbates the production of inflammatory cytokines production in LPS-stimulated BMDMs.

Next, macrophage depletion and reconstitution experiments were also performed in LPS-injected or *S. Typhimurium-*infected mice (**Figure 7D**). As shown in **Figure 7E**, macrophage-depleted (by clodronate liposomes treatment) mice reconstituted with LV-*Fto* (WT)-rescued mye*Fto* ^-/-^ BMDMs showed a markedly decreased survival rate and colon length compared with that of LV-Ctrl-rescued mye*Fto* ^-/-^ BMDMs (**Figures 7E, G**, red *vs.* yellow, **7H**). Furthermore, reconstitution with LV-*Fto* (S95A) rescued mye*Fto* ^-/-^ BMDMs exhibited more serious mortality (**Figures 7E, G**, green *vs.* red), elevated serum proinflammatory cytokines IL-1β/IL-6/TNF-α expression (**Figure 7F**) and contraction of the colon length (**Figure 7H**). We next performed HE analysis, more serious progressive pathological lesions including swelling and infiltration of inflammatory cells into the lamina propria and submucosa of the colon in *S. Typhimurium-*infected mice reconstituted with LV-*Fto* (S95A)-rescued mye*Fto* ^-/-^ BMDMs (**Figure 7I**, as indicated by black arrows in **7I**). Therefore, FTO O-GlcNAcylation mutation at Ser95 exacerbates the hyperinflammatory phenotype in LPS-treated or *S. Typhimurium-*infected mice, while FTO O-GlcNAcylation suppresses these hyperinflammation phenotypes in mice.3.8 O-GlcNAcylation of FTO increases *Socs1* m^6^A methylation and thus alleviates LPS-induced inflammatory responses and septic shock in mice.

**Figure 8:**
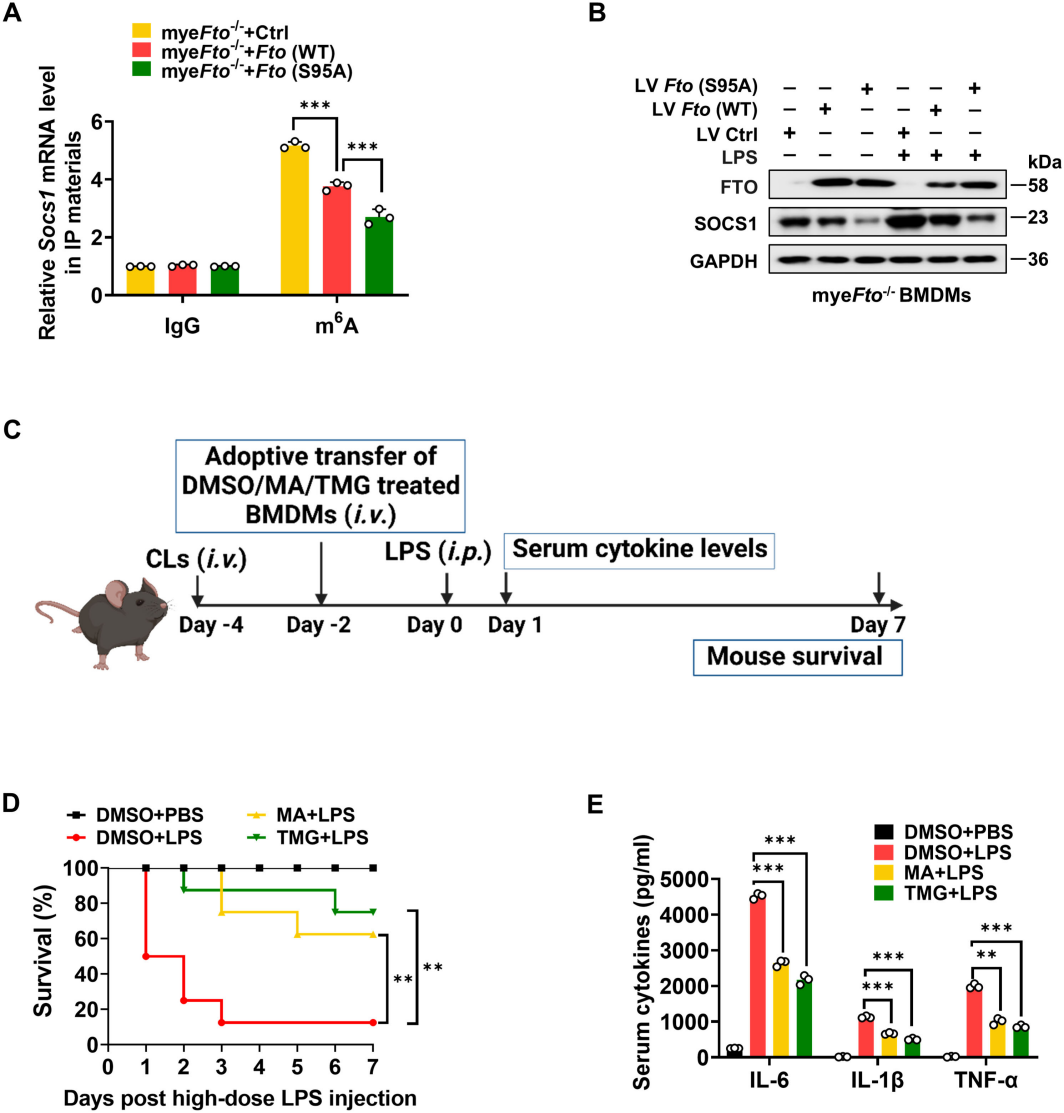
O-GlcNAcylation of FTO increases *Socs1* m^6^A methylation and thus alleviates LPS-induced inflammatory responses and septic shock in mice. **(A)** RIP-qPCR assays validating the LPS-induced m^6^A levels on *Socs1* transcript in LV*-*Ctrl/*Fto* (WT)/*Fto* (S95A)-infected mye*Fto* ^-/-^ mouse BMDMs. **(B)** Immunoblot analysis of SOCS1 expression in mye*Fto* ^-/-^ mouse BMDMs infected with LV-Ctrl/*Fto* (WT)/*Fto* (S95A) followed by PBS/LPS stimulation. **(C)** The illustration of macrophage depletion and reconstitution procedure. **(D)** Kaplan–Meier survival curves of macrophage-depleted mice reconstituted with mouse BMDMs treated with DMSO or MA or TMG, followed by PBS or LPS (25 mg/kg, mouse) challenge. Log-rank (Mantel-Cox) test was used to assess the statistical difference. 8 mice were in each group. **(E)** ELISA of serum IL-6/IL-1β/TNF-α concentrations from mice **(D)** injected with PBS or LPS (25 mg/kg, mouse) for 24 (h) GAPDH served as a loading control for **(B)**. Data are representative of three independent experiments. Data represent the mean ± SD with significance determined by one-way ANOVA followed by Tukey’s multiple comparison tests for **(A)**, and by one-way ANOVA followed by Dunnett multiple comparisons tests for **(E)**. **p<0.01; ***p<0.001.

It has been reported that m^6^A eraser FTO can mediate *Socs1* m^6^A demethylation (22). This previous study has identified *Socs1* mRNA as a top target of m^6^A in macrophages through unbiased global bioinformatic analyses based on m^6^A methylation sequencing (m^6^A-RIP-seq) and RNA sequencing (RNA-seq). They have also proved that LPS treatment induces *Socs1* m^6^A methylation and sustains SOCS1 induction by decreasing *Fto* mRNA expression, which maintains the negative feedback control of macrophage cytokine storm in sepsis. Therefore, we determined the effect of FTO O-GlcNAcylation on *Socs1* m^6^A methylation and SOCS1 induction. LV*-*Ctrl/*Fto* (WT)/*Fto* (S95A)-infected mye*Fto* ^-/-^ mouse BMDMs treated with LPS were harvested and lysed to m^6^A-RIP-qPCR. We observed that *Fto* (WT)-rescued mye*Fto* ^-/-^ mouse BMDMs showed decreased m^6^A methylation compared with the LV*-*Ctrl group (**Figure 8A**). Furthermore, *Fto* (S95A)-rescued mye*Fto* ^-/-^ mouse BMDMs showed lower m^6^A methylation compared with *Fto* (WT)-rescued mye*Fto* ^-/-^ mouse BMDMs (**Figure 8A**), suggesting that O-GlcNAcylation of FTO increases *Socs1* m^6^A methylation. We also observed that *Fto* (S95A)-rescued mye*Fto* ^-/-^ mouse BMDMs showed decreased SOCS1 protein levels compared with *Fto* (WT)-rescued mye*Fto* ^-/-^ BMDMs under LPS treatment (**Figure 8B**). These results indicate that FTO O-GlcNAcylation at the Ser95 site increases *Socs1* m^6^A methylation and is required to sustain SOCS1 protein induction.

Next, we further detected the effects of the OGA/FTO inhibitors on the inflammatory responses in LPS-treated mice (**Figure 8C**). Macrophage depletion and reconstitution with mouse BMDMs-treated with DMSO or FTO-inhibitor MA (58)/OGA-inhibitor TMG experiments were also performed in LPS-injected mice. MA or TMG-treated groups showed a higher survival rate than DMSO-treated BMDMs after the LPS challenge (**Figure 8D**, yellow *vs.* red; green *vs.* red). Accordingly, the serum proinflammatory cytokines levels from MA or TMG-treated groups were much lower compared to those in the DMSO-treated group (**Figure 8E**). Therefore, the promotion of O-GlcNAcylation by the OGA inhibitor or the inhibition of FTO by the FTO inhibitor alleviates LPS-induced hyperinflammation and septic shock in mice.

## 4 Discussion

Accumulating evidences have shown that FTO participates in multiple inflammatory reactions (22–26). FTO expression is downregulated by LPS stimulation in macrophages (22–24). However, the underlying mechanism of FTO downregulation during LPS stimulation remains elusive. O-GlcNAcylation plays an important role in numerous human diseases, particularly inflammatory conditions (27–30). However, the potential involvement of O-GlcNAcylation in FTO stability and function needs further clarification.

**Figure 9:**
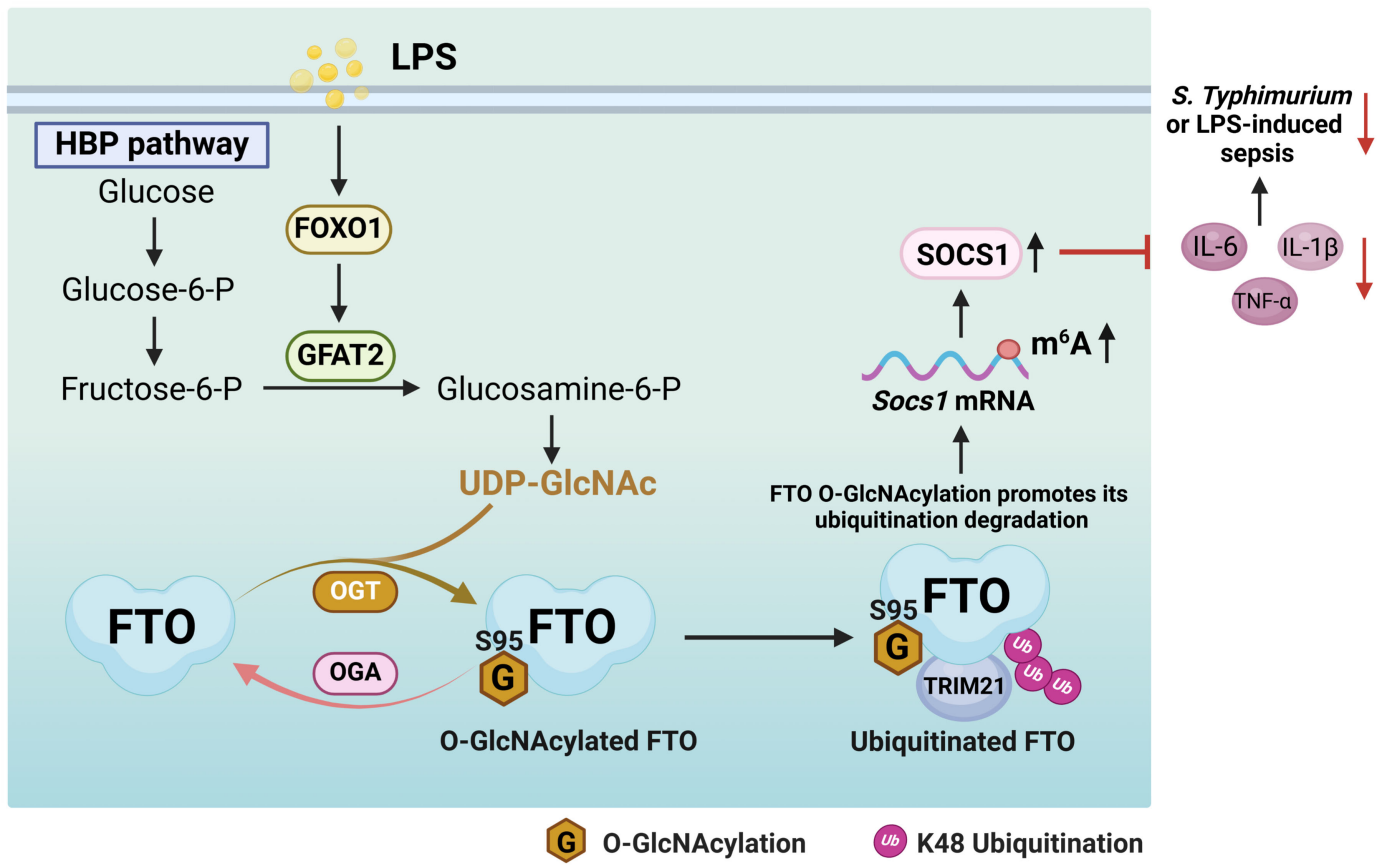
A proposed mechanism by which FTO O-GlcNAcylation promotes TRIM21-mediated FTO ubiquitination degradation to sustain the negative feedback control of macrophage inflammation. LPS enhances FTO O-GlcNAcylation through increasing the FOXO1-regulated GFAT2 expression in macrophages. FTO O-GlcNAcylation promotes TRIM21-mediated FTO K48-ubiquitination degradation, thus induces *Socs1* m^6^A methylation and sustains SOCS1 induction to suppress IL-1β/IL-6/TNF-α production in LPS-stimulated macrophages. FTO O-GlcNAcylation alleviates *S. Typhimurium* or LPS-induced sepsis.

Here we uncover that FTO undergoes OGT mediated-O-GlcNAcylation at the Ser95 site. LPS does not affect the protein expression levels of OGT and OGA, while LPS enhances FTO O-GlcNAcylation by increasing the FOXO1-regulated GFAT2 expression in macrophages. FTO O-GlcNAcylation facilitates TRIM21-mediated FTO ubiquitination and degradation, which induces *Socs1* m^6^A methylation and sustains SOCS1 induction to suppress inflammatory cytokines IL-1β/IL-6/TNF-α production in LPS-treated macrophages. FTO Ser95 site O-GlcNAcylation mutation aggravates *S. Typhimurium* or LPS-induced hyperinflammation and sepsis. Our findings reveal a previously unreported mechanism by which FTO O-GlcNAcylation acts as a negative regulator of inflammatory sepsis (**Figure 9**). Our study suggests that the promotion of FTO O-GlcNAcylation constitutes a regulation strategy for the intervention of the inflammatory processes in macrophages.

Previous studies have shown that *Socs1* and *Socs3* can target the Traf6 complex and inhibit signaling pathways associated with inflammation and innate immunity (59) LPS treatment induces *Socs1* m^6^A methylation and sustains SOCS1 induction by decreasing *Fto* mRNA expression, which maintains the negative feedback control of macrophage cytokine storm in sepsis (22). Our present research proves that LPS downregulates FTO protein expression by enhancing FTO O-GlcNAcylation-mediated ubiquitination degradation, which induces *Socs1* m^6^A methylation and sustains SOCS1 induction to suppress inflammation. These results indicate that LPS displays negative regulation of inflammation. However, LPS is usually deemed to cause the upregulation of pro-inflammatory mediators to promote immune responses (60), there are reports showing that LPS induces anti-inflammatory factors, such as *Socs1*, *Mirt2*, and *Il-10* (61–65). *Socs1* expression is rapidly induced by LPS and directly suppresses LPS-induced TLR4 inflammation signaling and endotoxin shock (66). LPS-induced long non-coding RNA (lncRNA) *Mirt2* functions as a checkpoint to prevent aberrant activation of inflammation (62). Consistently, our present study shows that LPS-induced FTO O-GlcNAcylation enhances *Socs1* m^6^A methylation to negatively regulate LPS-mediated inflammatory response. A previous report has also elucidated the molecular mechanism of LPS-induced downregulation of *Fto* at the transcriptional level (22). Based on our present data and previous reports, we propose that the negative feedback regulation mechanism of LPS may be mediated by both transcriptional level and post-translational modification, including *Fto* mRNA downregulation and FTO protein O-GlcNAcylation and ubiquitination degradation. Our study supports the existence of a cellular homeostasis mechanism of immunity for protection from excessive inflammation by FTO O-GlcNAcylation.

In the present study, we have analyzed four potential O-GlcNAcylation sites (S92, S95, S226, and S237) in FTO. The two potential O-GlcNAcylation sites (S92 and S95) are obtained by Co-IP and mass spectrometry analysis, and another two sites (S226 and S237) are predicted by O-GlcNAcylation sites prediction online databases: YinOYang 1.2 Server and OGTSite. We have mutated these four sites by substitutions of serine with alanine, respectively, and found that only the S95A mutation of FTO significantly decreased FTO O-GlcNAcylation levels among these four mutants and WT FTO (**Figure 2E**). Further functional analysis showed that the FTO (S95A) mutation increases FTO protein stability and aggravates *S. Typhimurium* or LPS-induced inflammatory response and sepsis, suggesting that the Ser95 is the critical functional O-GlcNAcylation site for FTO. A recent study reported that O-GlcNAcylation of FTO at the Ser173 site promotes myelodysplastic syndromes (MDS) and acute myeloid leukemia (AML) cell proliferation (67). Consistently, our data also showed that the FTO O-GlcNAcylation level was decreased but not completely abolished by the S95A mutation of FTO, indicating there are possibly other O-GlcNAcylation sites present in FTO, and the functions of these O-GlcNAcylation sites of FTO still need further investigation. It is also worth further investigating whether FTO O-GlcNAcylation is associated with other aberrant FTO-mediated m^6^A-related diseases, such as diabetes mellitus, hepatic fibrosis, melanoma, and other various malignancies by using more mouse models and clinical samples.

Our research shows that LPS does not affect the protein expression levels of OGT but increases its interaction with FTO. Correlatively, a previous report has proved that LPS stimulation had no detectable effect on the enzymatic activity of OGT in macrophages through OGT enzymatic assay (48). The possible explanation might be that LPS stimulation leads to the alteration of post-translational modifications of OGT itself that causes the increase of its substrate affinity with FTO. Future studies will be needed to explore whether the post-translational modifications of OGT increase its interaction with FTO during LPS stimulation.

GFAT, the enzyme that catalyzes the rate-limiting step of the HBP, exists as two isoforms (GFAT1 and GFAT2). A previous report has shown that LPS stimulation slightly affects the expression of GFAT1 but significantly increases the expression of GFAT2 in macrophages (48). Our study demonstrates that LPS stimulation enhances GFAT2 expression and increases FTO O-GlcNAcylation through FOXO1-regulated GFAT2 expression. Whether GFAT1 also makes some contributions to HBP flux in BMDMs upon LPS stimulation will need to be investigated in the future.

FOXO1 is a transcription factor that targets numerous genes to regulate cellular resistance, metabolism, cell-cycle progression, oxidative stress response, and apoptosis (68). The O-GlcNAcylation levels of the proteins (such as FTO) depend on either OGT/OGA expression/enzymatic activities or UDP-GlcNAc incorporation. Our data demonstrates that LPS increases FTO O-GlcNAcylation through FOXO1-regulated GFAT2 expression, thereby increasing UDP-GlcNAc incorporation into FTO. Whether FOXO1 affects FTO O-GlcNAcylation through other pathways (such as the post-translational modifications of OGT/OGA), except GFAT2 and UDP-GlcNAc synthesis, will also need investigation.

O-GlcNAcylation has been shown to have both pro-inflammatory and anti-inflammatory effects in macrophages. For example, elevated O-GlcNAcylation of signaling proteins of NF-κB or STAT3 pathways accelerates the inflammatory response (69, 70), but increased O-GlcNAcylation of RIPK3 or mSin3A was shown to suppress macrophage inflammation upon LPS stimulation (28, 71). It has also been reported that GlcN displays a protective effect on sepsis potentially through modulation of O-GlcNAcylation of nucleocytoplasmic proteins and enzymatic regulation of O-GlcNAcylation potentially plays an important role in LPS-mediated inflammation and recovery (72). Based on our results and other studies, we propose that the functions of O-GlcNAcylation on different proteins might be different. FTO O-GlcNAcylation at Ser95 is important for anti-inflammatory effects.

Crosstalk between different types of protein post-translational modifications determines a wealth of biological processes. It is known that O-GlcNAcylation has extensive crosstalk with other post-translational modifications, such as phosphorylation, ubiquitination, acetylation, methylation, and hydroxylation, which is important for regulating target protein function (47, 73, 74). Previous studies identified that O-GlcNAcylation regulates protein ubiquitination through interplay with phosphorylation, recruiting ubiquitinases/deubiquitinases, and other unknown mechanisms (36). Interestingly, our findings reveal that promotion of total O-GlcNAcylation modification by OGA inhibitor TMG can suppress FTO phosphorylation levels, while mutation of FTO O-GlcNAcylation at the Ser95 site does not affect FTO phosphorylation levels (**Figure 2I**). Importantly, O-GlcNAcylation of FTO recruits the E3 ubiquitinase TRIM21 and results in an intensive interaction between TRIM21 and FTO, thereby promoting the K48 ubiquitination degradation of FTO.

Remarkably, previous studies have identified that O-GlcNAcylation modulated the stability of many proteins either positively or negatively (36, 75, 76). O-GlcNAcylation of sine oculis homeobox homolog 1 (SIX1) enhances its stability by inhibiting the ubiquitination degradation of SIX1, which promotes hepatocellular carcinoma proliferation (75). Oppositely, O-GlcNAcylation of ryanodine receptor 1 (RYR1) increases its K48-linked ubiquitination and proteasomal degradation to decrease RYR1 abundance (76). Our present study demonstrates that FTO O-GlcNAcylation promotes its ubiquitination and shows negative regulation of FTO protein stability through the K48 ubiquitin pathway. The modulation of O-GlcNAcylation on the stability or degradation of different proteins represents a context-dependent regulation form.

Modulation of O-GlcNAcylation holds potential therapeutic value, since it only modulates post-translational modifications of proteins, but does not affect the synthesis of cellular protein and nucleic acid. We observed that enhancing O-GlcNAcylation of FTO using an OGA inhibitor (TMG) decreased LPS-induced TNF-α/IL-1β/IL-6 inflammation and reduced septic mouse mortality, which may constitute a protective mechanism to limit exacerbated inflammation in acute stress conditions such as LPS-induced sepsis. The OGA inhibitor TMG (20 μM) has similar or seemingly better therapeutic effects on LPS-induced septic shock than FTO inhibitor MA (100 μM). TMG holds extremely high selectivity for human O-GlcNAcase (45). Increasing O-GlcNAc levels of tau protein through TMG treatment could block the accumulation of toxic tau in Alzheimer’s disease (77). We propose that the utilization of OGA inhibitors or a combination of FTO and OGA inhibitors might also constitute a better treatment strategy for the intervention of endotoxin-induced inflammation. The small molecules, named O-GlcNAcylation TArgeting Chimeras (OGTACs) have also been developed, which selectively enable specific protein O-GlcNAcylation in living cells (78). We postulate that targeted upregulating FTO O-GlcNAcylation by OGTACs might be a promising anti-inflammatory strategy. In addition, other cell types and mouse models (such as human mononuclear macrophages, cecum ligation and puncture mouse sepsis models) will be needed for verification.

In summary, our present study reveals three previously unrecognized findings: First, FTO undergoes O-GlcNAcylation specifically at the Ser95 site. Second, LPS does not affect the expression of OGT and OGA, while LPS increases FTO O-GlcNAcylation by increasing the FOXO1-regulated GFAT2 expression in macrophages. Third, FTO O-GlcNAcylation promotes TRIM21-mediated FTO ubiquitination degradation, which induces *Socs1* m^6^A methylation and sustains SOCS1 induction to maintain the negative feedback control of macrophage inflammatory cytokine storm in sepsis. Our findings clarify the regulatory network that exists among LPS-induced FTO O-GlcNAcylation-ubiquitination-inflammatory response. Enhancement of the FTO O-GlcNAcylation level might serve as a potential therapeutic strategy for combating endotoxin-induced inflammation or other FTO abnormal expression-associated diseases.

## Data Availability

The datasets presented in this study can be found in online repositories. The names of the repository/repositories and accession number(s) can be found below: PXD047698 and PXD047256 (PRIDE).
