# Supplementary material for: FTO O-GlcNAcylation promotes TRIM21-mediated FTO ubiquitination degradation to sustain the negative feedback control of macrophage inflammation
Source: Front Immunol. 2025 Jun 26;16:1593243. doi: 10.3389/fimmu.2025.1593243 (PMC12240770; doi:10.3389/fimmu.2025.1593243)
Supplement: Supplementary file 1 [file DataSheet1.pdf]

Supplementary Material

Supplementary Material for
FTO O-GlcNAcylation promotes TRIM21-mediated FTO ubiquitination degradation to sustain the negative feedback control of macrophage inflammation

The PDF file includes:

Supplementary Figures: 1 to 4
Legends for Supplementary Figures: 1 to 4
Supplementary Tables: 1 to 7

Supplementary Figures

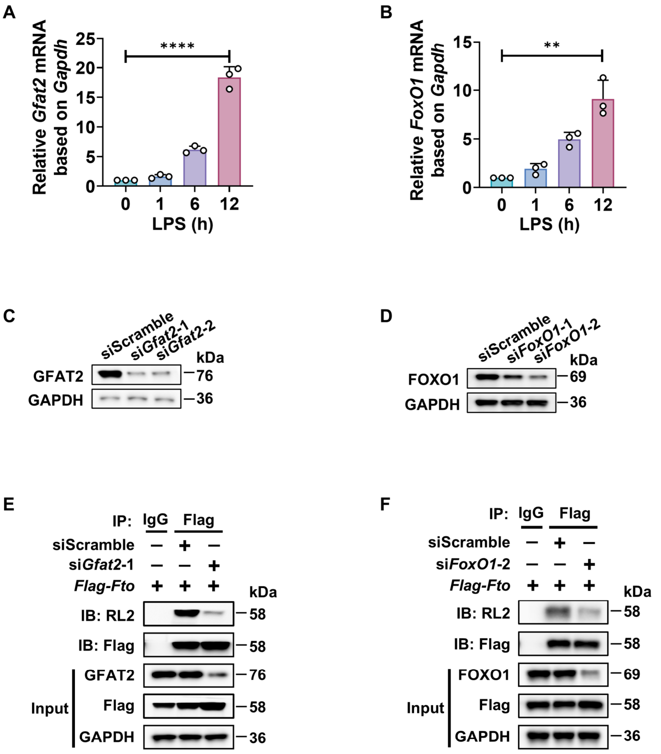

Supplementary Figure 1. *Gfat2*/*FoxO1* knockdown decreased FTO O-GlcNAcylation. (A, B) RT-qPCR quantitation of *Gfat2* **(A)** or *FoxO1* **(B)** transcripts in BMDMs treated with LPS. **(C, D)** Immunoblot analysis of si*Gfat2*-1/si*Gfat2*-2 **(C)** and si*FoxO1*-1/si*FoxO1*-2 **(D)** silencing efficiencies in BMDMs. **(E, F)** Co-IP and immunoblot analysis of the effects of *Gfat2* **(E)** and *FoxO1* **(F)** silencing on FTO O-GlcNAcylation. RAW264.7 cells were transfected with Flag-*Fto* and si*Gfat2*-1 **(E)**/si*FoxO1*-2 **(F)** for 48 h and subjected to Co-IP with an anti-Flag and immunoblot with Abs against GFAT2, FOXO1, RL2, and Flag, respectively. GAPDH served as a loading control for **(C-F)**. Data are representative of three independent experiments. Data represent the mean ± SD with significance determined by one-way ANOVA followed by the Dunnett multiple comparison tests for **(A, B)**. $^{**}p<0.01$; $^{****}p<0.0001$.

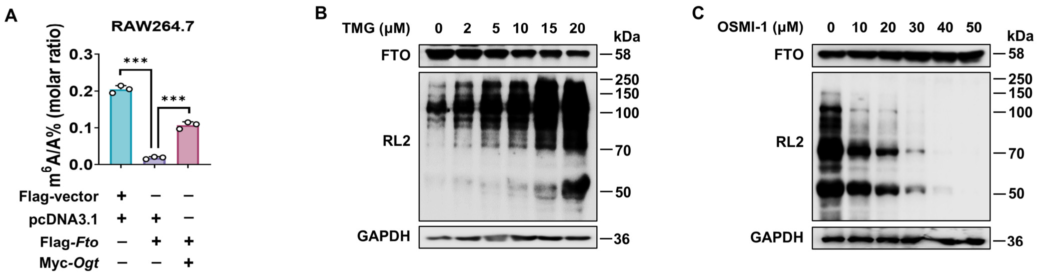

Supplementary Figure 2. FTO O-GlcNAcylation downregulates the protein expression of FTO. (A) LC-MS/MS detection of m^6A/A (%) of total RNA in individual RAW264.7 cells transfected with Flag-*Fto* or Myc-*Ogt*. **(B, C)** Immunoblot analysis of the FTO expression in RAW264.7 cells treated with TMG **(B)** or OSMI-1 **(C)** for 24 h. GAPDH served as a loading control for **(B, C)**. Data are representative of three independent experiments. Data represent the mean ± SD with significance determined by one-way ANOVA followed by the Dunnett multiple comparison tests for **(A)**. ***p <0.001.

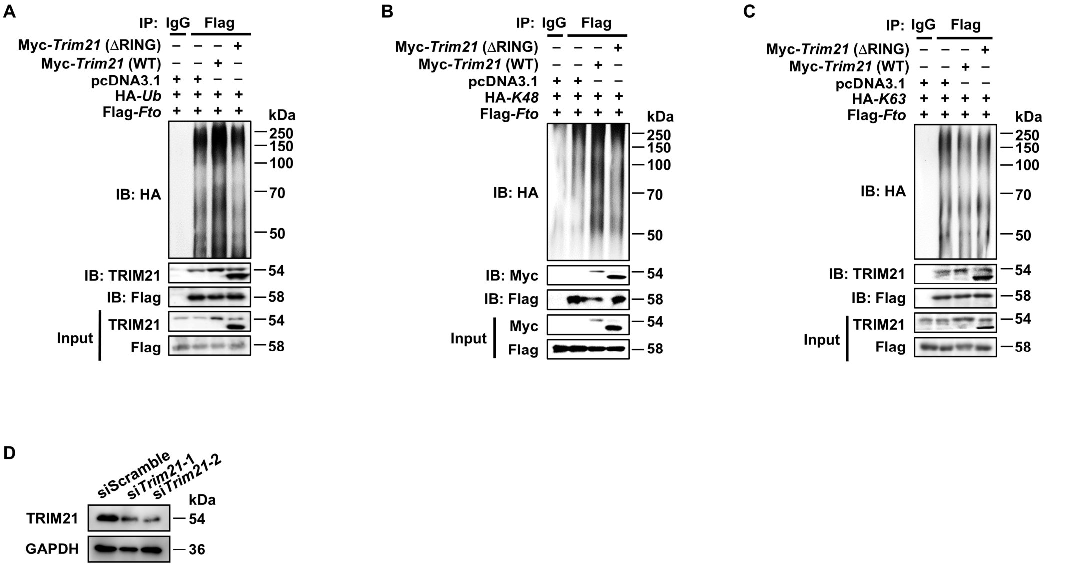

Supplementary Figure 3. TRIM21 mediates ubiquitination of FTO. (A-C) Co-IP and immunoblot analysis of the effects of TRIM21 on FTO ubiquitination. HEK293T cells were co-transfected with Flag-*Fto* and HA-*Ub* **(A)**/*K48* **(B)**/*K63* **(C)** plus Myc-*Trim21* (WT) or Myc-*Trim21* (DRING) mutant vector. **(D)** Immunoblot analysis of si*Trim21*-1/si*Trim21*-2 silencing efficiencies in BMDMs. Data are representative of three independent experiments.

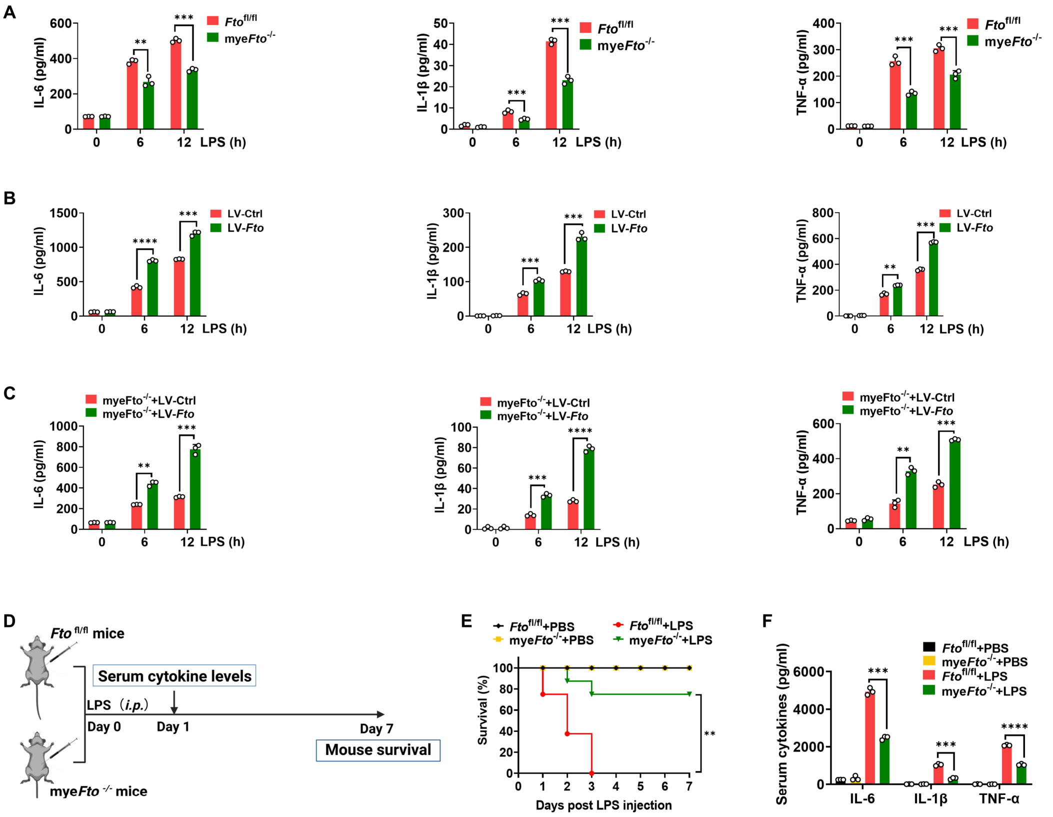

Supplementary Figure 4. Myeloid-specific *Fto* deletion suppresses the inflammatory response during LPS stimulation and FTO promotes macrophage inflammation. (A) ELISA of supernatants IL-6/IL-1b/TNF-a concentrations of *Fto*$^{fl/fl}$ and mye*Fto*$^{-/-}$ mouse BMDMs treated with LPS for the indicated periods. **(B)** The wild-type BMDMs were infected with *Fto*-lentivirus (LV-*Fto*) or LV-Ctrl and then treated with LPS for the indicated time. Measurement of supernatants IL-6/IL-1b/TNF-a concentrations of the BMDMs by ELISA. **(C)** The mye*Fto*$^{-/-}$ BMDMs were infected with LV-*Fto* or LV-Ctrl and then treated with LPS for the indicated time. Measurement of supernatants IL-6/IL-1b/TNF-a concentrations of mye*Fto*$^{-/-}$ BMDMs by ELISA. **(D)** Procedure for LPS-induced sepsis experiment in *Fto*$^{fl/fl}$ and mye*Fto*$^{-/-}$ mice. **(E)** Kaplan–Meier survival curves of *Fto*$^{fl/fl}$ and mye*Fto*$^{-/-}$ mice following PBS or LPS (25 mg/kg, mouse) treatment. Log-rank (Mantel-Cox) test was used to assess the statistical difference. 8 mice were in each group. **(F)** ELISA of serum IL-6/IL-1b/TNF-a concentrations in *Fto*$^{fl/fl}$ and mye*Fto*$^{-/-}$ mice after PBS or LPS (25 mg/kg) challenge for 24 h. Data are representative of three independent experiments. Data represent the mean ± SD with significance determined by two-tailed unpaired Student's t-test for **(A-C)**, and by one-way ANOVA followed by Tukey's multiple comparison tests for **(F)**. ***p*<0.01; ****p*<0.001; *****p*<0.0001.

Supplementary Tables

Supplementary Table 1. Primers used in RT-qPCR

Genes	Forward (5'-3')	Reverse (5'-3')
Wtap	ACAGGCAAAAAGCTAATGGCG	CAACTGCTGTCGTGTCTCCT
Mettl3	GTGGCTTTTCATCTTGGCTCTATC	CAGTAGGCACGGGACTATCA
Mettl14	ACACTGGACACAGACCTCAGA	TCATCCCAGGTCCAGCATTTC
Fto	CCTCAATGACTCAGACGATGG	GAGCACCGCATTTGTCATGC
Alkbh5	ATTGCCACCCAGCTATGCTT	AGACCGCCGGTTTTCTTCTT
Gfat2	GTATGATTGGCCGACCCTGG	ATGCTAGCCGGAGAGCTGAA
ChIP-*Gfat2*	AAGTGCTCTACTACTGAGCTAAGTCC	GCTACCACTGCTGAGGAATCTAAC
FoxO1	CCCAGGCCGGAGTTTAACC	GTTGCTCATAAAGTCGGTGCT
Socs1	CTGCGGCTTCTATTGGGGAC	AAAAGGCAGTCGAAGGTCTCG
Gapdh	CAGGAGAGTGTTTCCTCGTCC	GATGGGCTTCCCGTTGATGA

Supplementary Table 2. Antibodies used in this study

Antibodies	Source	Identifier
FTO	Abcam	Cat. No. ab92821, RRID: AB_10565042
O-GlcNAc (RL2)	Abcam	Cat. No. ab2739, RRID: AB_303264
OGT	Cell Signaling Technology	Cat. No. 24083, RRID: AB_2716710
OGA	ABclonal Biotech	Cat. No. A24124, RRID: AB_3668909
FOXO1	ABclonal Biotech	Cat. No. A2934, RRID: AB_2764748
GFAT2	ABclonal Biotech	Cat. No. A15374, RRID: AB_2762279
TRIM21	ABclonal Biotech	Cat. No. A18027, RRID: AB_2861823
KCTD10	Proteintech	Cat. No. 27279-1-AP, RRID: AB_2880827
p-Ser/Thr	ABclonal Biotech	Cat. No. AP1067, RRID: AB_2863939
Rabbit anti-Flag	ABclonal	Cat. No. AE092, RRID: AB_2940847
Mouse anti-Myc	Proteintech	Cat. No. 60003-2-Ig RRID: AB_2734122
Mouse anti-HA	Abcam	Cat. No. ab18181, RRID: AB_444303
Ubiquitin	Proteintech	Cat. No. 10201-2-AP, RRID: AB_671515
K48-ubiquitin	Abcam	Cat. No. ab140601, RRID: AB_2783797
K63-ubiquitin	Abcam	Cat. No. ab179434, RRID: AB_2895239
SOCS1	ABclonal Biotech	Cat. No. A7754, RRID: AB_2772339
m^6A	ABclonal Biotech	Cat. No. A19841, RRID: AB_2862753
GAPDH	Santa Cruz Biotechnology	Cat. No. sc-365062, RRID: AB_10847862
Rabbit IgG	Cell Signaling Technology	Cat. No. 3900, RRID: AB_1550038
Mouse IgG	Proteintech	Cat. No. 66360-1-Ig, RRID: AB_2827991
Alexa Fluor 488-anti-mouse	Cell Signaling Technology	Cat. No. 4408, RRID: AB_2130165
Alexa Fluor 594-anti-rabbit	Cell Signaling Technology	Cat. No. 8889, RRID: AB_2716249
HRP conjugate anti-Mouse IgG	Proteintech	Cat. No. SA00001-1, RRID: AB_2722565
HRP conjugate anti-Rabbit IgG	Proteintech	Cat. No. SA00001-2, RRID: AB_2722564

Supplementary Table 3. E3 ubiquitin ligases from mass spectrometry

Protein/Accession	Description	Score	Coverage
TRIM21/Q3U7K7	RING-type E3 ubiquitin-protein ligase	4.02	3.89
KCTD10/Q922M3	The substrate-specific adapter of E3 ligase	8.81	16.19

Supplementary Table 4. All siRNAs used in this study

Genes	Sense (5'-3')	Antisense (5'-3')
si*Gfat2*-1	CCGAGAGCUCCAAGUUUGCAUAUAA	UUAUAUGCAAACUUGGAGCUCUCGG
si*Gfat2*-2	CAAAGGCUACGAGUUUGAGUCAGAA	UUCUGACUCAAACUCGUAGCCUUUG
si*FoxO1*-1	GAAAGAGUUCUUGGUGGAUGCUCAA	UUGAGCAUCCACCAAGAACUCUUUC
si*FoxO1*-2	CAAGUUACGGAGGAUUGAACCAGUA	UACUGGUUCAAUCCUCCGUAACUUG
si*Trim21*-1	CAACAGUUUCUGCUCCGAATT	UUCGGAGCAGAAACUGUUGTT
si*Trim21*-2	GGACAUGUUGGGUUCAUAUTT	AUAUGAACCCAACAUGUCCTT

Supplementary Table 5. Primer sequences for molecular clonings

Primer names	Primer sequences (5'-3')
pFlag-CMV-*Fto*	F: TACAAAGACGATGACGACAAGCTTATGAAGCGCGTCCAGACCGCGG<br>R: CGATATCAGATCTATCGATGAATTCCTAGGATCTTGCTTCCAGCAG
pFlag-CMV-*Fto*-N	F: CCAAGCTTATGAAGCGCGTCCAGACCGCGGAGGA<br>R: CGGAATTCCTACTCTGCCACACGGTGAGTGGAACTAAAC
pFlag-CMV-*Fto*-C	F: CCAAGCTTATGTGCTCAACAGGCACCTTGGAT<br>R: CGGAATTCCTAGGATCTTGCTTCCAGCAGCTGGCCTCT
pFlag-CMV-*Fto* (S95A)	F2: GCTCACCCCAGTGGCTCGCATCCTCAT<br>R2: ATGAGGATGCGAGCCACTGGGGTGAGC
pFlag-CMV-*Fto* (T92A)	F2: CAAAGATGTGCTGCCCCAGTGTCTCGC<br>R2: GCGAGACACTGGGGCAGCACATCTTTG
pFlag-CMV-*Fto* (S226A)	F2: GAAGATGGCGGTGGCCTGGCATCACGAT<br>R2: ATCGTGATGCCAGGCCACCGCCATCTTC
pFlag-CMV-*Fto* (S237A)	F2: CTGGTGGACAGGGCAGCCGTGGCAGT<br>R2: ACTGCCACGGCTGCCCTGTCCACCAG
pFlag-CMV-*Fto* (K213R)	F2: ATGCCCTACTTGAGAGAGGAGCCCTAT<br>R2: ATAGGGCTCCTCTCTCAAGTAGGGCAT
pcDNA3.1-Myc-His-*Ogt*	F: TTGGATCCATGGCGTCTTCCGTGGGCAACGTG<br>R: CCAAGCTTGGCTGACTCGGTGACTTCAACAGGCTTA
pcDNA3.1-Myc-His-*Ogt* (K908A)	F2: ATTTTCTCACCTGTGGCTCCTGCAGAGGAGCATGTCAGGAGAG<br>R2: ACCTCTCCTGACATGCTCCTCTGCAGGAGCCACAGGTGAGAAA
pcDNA3.1-Myc-His-*Ogt*-F1	F: CACCACACTGGACTAGTGGATCCACAGAATCCCCTTCTAGCAG<br>R: CCAAGCTTGGCTGACTCGGTGACTTCAACAGGCTTA
pcDNA3.1-Myc-His-*Ogt*-F2	F: AGAATCCCCTTCTAGCAGAAAATCCTGATTTGTACTGTGTTCGCAGT<br>R: CACTGCGAACACAGTACAAATCAGGATTTTCTGCTAGAAGGGGATT
pcDNA3.1-Myc-His-*Ogt*-F3	F: GATTTGTACTGTGTTCGCAGTGACCCTTGACCCAAATTTTCTGGATGC<br>R: CCAGAAAATTTGGGTCAAGGGTCACTGCGAACACAGTACAAATCAG
pcDNA3.1-Myc-His-*Gfat2*	F: AGCGTTTAAACGGGCCCTCTAGAATGTGCGGAATCTTTGCCTACAT<br>R: AGCACAGTGGCGGCCGCTCGAGATTCCACAGTGACAGACTTGGCT
pGL3-*Gfat2*-promoter-luc	F: TGCTAGCCCGGGCTCGAGTGTGTGTGTGTGTGT<br>R: ACCGGAATGCCAAGCTTCCCTTATAGCCAGCCAG
pGL3-*Gfat2* promoter site 1-mutagenesis-luc	F: GATATTTCATGTAACGACACACACACACACAC<br>R: GTGTGTGTGTGTGTGTCGTTACATGAAATATC
pGL3-*Gfat2* promoter site 2-mutagenesis-luc	F: GATAAGTAGAATTCAATTGGAACCGAGACCAAGGAAGAGCTTGCATAG<br>R: CTATGCAAGCTCTTCCTTGGTCTCGGTTCCAATTGAATTCTACTTATC
pcDNA3.1-Myc-His-*FoxO1*	F: GCTAGCGTTTAAACGGGCCCTCTAGAATGGCCGAAGCGCCCCAGGTG<br>R: CACAGTGGCGGCCGCTCGAGAGCCTGACACCCAGCTGTGTGTTGTAG
pcDNA3.1-Myc-His-*Trim21*	F: CCCTCGAGGATGTCACCCTCTACAACCTC<br>R: CGGAATTCCGCATCTTTAGTGGACAGAGCTT
pcDNA3.1-Myc-His-*Trim21* (Mut)	F: CCCTCGAGGCTGCTCCGAAACCTCAGGCCC<br>R: CGGAATTCCGCATCTTTAGTGGACAGAGCTT
pLV-*Fto* (WT)	F: AGAAGATTCTAGAGCTAGCGAATTCATGAAGCGCGTCCAGACCGCGG<br>R: CGCAGATCCTTCGCGGCCGCGGATCCCTAGGATCTTGCTTCCAGCAG
pLV-*Fto* (S95A)	F2: GCTCACCCCAGTGGCTCGCATCCTCAT<br>R2: ATGAGGATGCGAGCCACTGGGGTGAGC

Supplementary Table 6. Plasmids used in this study

Recombinant plasmids	SOURCE	IDENTIFIER
pcDNA3.1-HA-Ub (WT)	Drs. Xuetao Cao and Baoxue Ge	N/A
pcDNA3.1-HA-Ub (K6)	Drs. Xuetao Cao and Baoxue Ge	N/A
pcDNA3.1-HA-Ub (K11)	Drs. Xuetao Cao and Baoxue Ge	N/A
pcDNA3.1-HA-Ub (K27)	Drs. Xuetao Cao and Baoxue Ge	N/A
pcDNA3.1-HA-Ub (K29)	Drs. Xuetao Cao and Baoxue Ge	N/A
pcDNA3.1-HA-Ub (K33)	Drs. Xuetao Cao and Baoxue Ge	N/A
pcDNA3.1-HA-Ub (K48)	Drs. Xuetao Cao and Baoxue Ge	N/A
pcDNA3.1-HA-Ub (K63)	Drs. Xuetao Cao and Baoxue Ge	N/A

Supplementary Table 7. All Reagents used in this study

Reagent	Source	Identifier
Lipopolysaccharide (LPS)	Sigma-Aldrich	Cat. No. L2630
GalNAz	Ruixibio	Cat. No. R-C-1225
GlcN	Sigma-Aldrich	Cat. No. G4875
UDP-GlcNAc	Sigma-Aldrich	Cat. No. U4375
DAPI	Sigma-Aldrich	Cat. No. D9542
Fetal bovine serum (FBS)	Gibco	Cat. No. 10099141
M-CSF	Peprotech	Cat. No. 315-02-50
Cycloheximide (CHX)	MCE	Cat. No. HY-12320
MG-132	MCE	Cat. No. HY-13259
3-Methyladenine (3-MA)	MedChemExpress	Cat. No. HY-19312
Chloroquine (CQ)	MedChemExpress	Cat. No. HY-17589A
Thiamet G (TMG)	ApexBio	Cat. No. B2048
OSMI-1	Sigma-Aldrich	Cat. No. SML1621
Meclofenamic acid (MA)	MCE	Cat. No. HY-117275
RNase Inhibitor	Takara	Cat. No. 2313B
Nuclease P1	Sigma-Aldrich	Cat. No. N8630
SAP	NEB	Cat. No. M0371S
Protein-A/G magnetic beads	MCE	Cat. No. HY-K0202
NEOFECT™ DNA transfection reagent	Neofect Biotech	Cat. No. TF201201
Lipofectamine 2000	Thermo Fisher	Cat. No. 12566014
PMSF	Roche	Cat. No. 10837091001
TRIzol Reagent	Invitrogen	Cat. No. 15596026
ReverTra Ace® qPCR RT Kit	Toyobo Life Science	Cat. No. FSQ-101
SYBR® Green Realtime PCR Master Mix	Toyobo Life Science	Cat. No. QPK-201
Liposomes and clodronate liposomes (CLs)	FormuMax	Cat. No. F70101C-A
Mouse TNF-α ELISA kit	Dakewe Biotech	Cat. No. 1217202
Mouse IL-6 ELISA kit	Dakewe Biotech	Cat. No. 1210602
Mouse IL-1β ELISA kit	Dakewe Biotech	Cat. No. 1210122
**Cell lines**		
RAW264.7	The China Center for Type Culture Collection (CCTCC)	Cat. No. GDC0143
HEK293T	The China Center for Type Culture Collection (CCTCC)	Cat. No. GDC0187
